# Very important pharmacogenetic variants landscape and potential clinical relevance in the Zhuang population from Yunnan province

**DOI:** 10.1038/s41598-024-58092-w

**Published:** 2024-03-29

**Authors:** Yujie Li, Yanting Chang, Yan Yan, Xiaoya Ma, Wenqian Zhou, Huan Zhang, Jinping Guo, Jie Wei, Tianbo Jin

**Affiliations:** 1 Key Laboratory of Resource Biology and Biotechnology in Western China (Northwest University), Ministry of Education, School of Life Sciences, Northwest University, #229 North TaiBai Road, Xi’an, 710069 Shaanxi China; 2 College of Life Science, Northwest University, Xi’an, 710127 China; 3 Provincial Key Laboratory of Biotechnology of Shaanxi Province, Northwest University, Xi’an, 710069 Shaanxi China

**Keywords:** Very important pharmacogene variant, Zhuang population, Single nucleotide variants, Potential clinical relevance, Personalized administration, Genetics, Risk factors

## Abstract

The gradual evolution of pharmacogenomics has shed light on the genetic basis for inter-individual drug response variations across diverse populations. This study aimed to identify pharmacogenomic variants that differ in Zhuang population compared with other populations and investigate their potential clinical relevance in gene-drug and genotypic-phenotypic associations. A total of 48 variants from 24 genes were genotyped in 200 Zhuang subjects using the Agena MassARRAY platform. The allele frequencies and genotype distribution data of 26 populations were obtained from the 1000 Genomes Project, followed by a comparison and statistical analysis. After Bonferroni correction, significant differences in genotype frequencies were observed of *CYP3A5* (rs776746), *ACE* (rs4291), *KCNH2* (rs1805123), and *CYP2D6* (rs1065852) between the Zhuang population and the other 26 populations. It was also found that the Chinese Dai in Xishuangbanna, China, Han Chinese in Beijing, China, and Southern Han Chinese, China showed least deviation from the Zhuang population. The Esan in Nigeria, Gambian in Western Division, The Gambia, and Yoruba in Ibadan, Nigeria exhibited the largest differences. This was also proved by structural analysis, Fst analysis and phylogenetic tree. Furthermore, these differential variants may be associated with the pharmacological efficacy and toxicity of Captopril, Amlodipine, Lisinopril, metoclopramide, and alpha-hydroxymetoprolol in the Zhuang population. Our study has filled the gap of pharmacogenomic information in the Zhuang population and has provided a theoretical framework for the secure administration of drugs in the Zhuang population.

## Introduction

Adverse drug reactions (ADRs) constitute a significant contributor to morbidity and mortality, ranking among the top 10 leading causes of death and disease in developed nations^1,2^. The characteristics of ADRs exhibit variability contingent upon factors such as genotype, age, gender, population, pathology, drug type, route of administration, and drug interaction^3,4^. According to Ingelman-Sundberg, genetic factors may account for approximately 10% to 20% of the occurrence of ADRs^5^. Genetic factors have been found to significantly influence pharmacokinetics, pharmacodynamics, and susceptibility to allergic reactions, resulting in changes in both local and systemic drug exposure and/or drug target functionality, ultimately impeding drug responses^6^. Recent investigations have elucidated the genetic underpinnings of ADRs^7^, thereby highlighting the close association between genetic factors and drug response.

Pharmacogenomics, as a key area of precision medicine, is the use of genomic and other “omic” information to personalize drugs selection and administration to avoid ADRs and maximize drugs therapeutic efficacy^8,9^. Pharmacogenomics accounted for 80% of the variations in drug treatment and safety. More than 400 genes were found to be involved in drug metabolism, and around 200 drug genes were linked to ADRs. It has been shown that substantial differences in distribution and frequencies of single nucleotide variants (SNVs) worldwide affect the key genes involved in drug absorption, distribution, metabolism, and elimination of abnormalities^10^. SNVs were a vast resource of genetic variation in humans, resulting in phenotypic differences among individuals^11,12^. The Pharmacogenetics and Pharmacogenomics Knowledge Base (PharmGKB; http://www.pharmgkb.org) that collects, organizes, and disseminates information on the impact of genetic variations in humans on drug responses. It provides free clinical-related information, including dosing guidelines, annotated drug labels, potentially viable gene-drug associations, and genotype–phenotype relationships^13^.

In recent years, numerous researchers have investigated very important pharmacogene (VIP) variants in ethnic minorities in China, such as the Tibetans^14^ and Lahu^15^. According to the 7th National Census, the Zhuang population totaled 15,721,956, ranking second only to the Han Chinese among the 56 populations. They are widespread in China’s Yunnan and Guizhou provinces, mainly in the Guangxi Zhuang Autonomous Region. Over a long period of time, they have developed customs and cultures with their own ethnic characteristics. However, we still have limited information on pharmacogenetic variants in the Zhuang population.

In this study, the VIP variants selected were derived from the PharmGKB, the SNP database of National Center for Biotechnology Information (NCBI, http://www.ncbi.nlm.nih.gov/SNP/), and the International HapMap Project (http://www.hapmap.org/), in addition to relevant pharmacogenomics literature. Then, the allele and genotype frequencies of the Zhuang population were compared with those of 26 other populations to obtain significant differences in SNVs after genotyping 200 unrelated Zhuang subjects from Yunnan province. The results of this study may complement current pharmacogenomics data of the Zhuang population, providing a theoretical basis for the safe use of drugs and predicting certain diseases in the Zhuang population.

## Materials and methods

### Study subjects

In total, 200 unrelated Zhuang subjects (110 females and 90 males) were recruited from Wen Shan in the Yunnan Province of China. The sample size and the proportion were determined using G*Power 3.1.9.2 software^16^. The participants were healthy based on their medical history and physical examination. Additionally, they had at least three generations of Zhuang ancestry, while none of the other populations had any known ancestral background. Subjects with chronic diseases, infectious diseases, drug or alcohol abuse, severe heart, liver or kidney dysfunction, immune disorders, pregnancy, and lactation were excluded. The informed consent forms have been signed by all subjects. According to the study protocol approved by the Clinical Research Ethics Committee of Northwest University, 5 mL of peripheral blood was collected from each subject and stored at 4 °C for 24 h.

### Variants selection and genotyping

Through an extensive literature review on drug metabolism and toxicity, we identified 24 genes associated with these phenomena. By utilizing resources such as the PharmGKB database, the SNP database of NCBI, and the International HapMap Project, in addition to relevant pharmacogenomics literature, we selected variants linked to drug therapy responsiveness. A preliminary screening identified 59 variants. However, only homozygous genotypes were observed for 11 of these variants, making it impossible to compare the distribution and differences in genotype frequency. Consequently, these 11 variants were excluded from our analysis, leaving 48 variants for further investigation.

Genomic DNA was extracted from participants’ peripheral blood using GoldMag-Mini Whole Blood Genomic DNA Purification Kit (GoldMag Ltd., Xi’an, China). The concentration of genomic DNA was measured using NanoDrop 2000C spectrophotometer (Thermo Scientific, Waltham, MA, USA). Subsequently, multiplexed SNV MassEXTEND assays were designed using Agena MassARRAY Assay Design 4.0 software (San Diego, California, USA), which allowed for the design of PCR primers for the selected VIP variants. Agena MassARRAY RS1000 (San Diego, California, USA) was able to genotype the 48 VIP variants according to the manufacturer's instructions^17^. Finally, the data of SNV genotypes were collected and managed using Agena Typer 4.0 software^18^, as mentioned in previous studies.

### Populations variation data

We downloaded the genotype data from the 1000 Genomes website (https://www.internationalgenome.org/). The 26 populations included: (1) Chinese Dai in Xishuangbanna, China (CDX); (2) Han Chinese in Beijing, China (CHB); (3) Southern Han Chinese, China (CHS); (4) Japanese in Tokyo, Japan (JPT); (5) Kinh in Ho Chi Minh City, Vietnam (KHV); (6) African Caribbeans in Barbados (ACB); (7) African Ancestry in Southwest USA (ASW); (8) Esan in Nigeria (ESN); (9) Gambian in Western Division, The Gambia (GWD); (10) Luhya in Webuye, Kenya (LWK); (11) Mende in Sierra Leone (MSL); (12) Yoruba in Ibadan, Nigeria (YRI); (13) Colombian in Medellin, Colombia (CLM); (14) Mexican Ancestry in Los Angeles, California (MXL); (15) Peruvian in Lima, Peru (PEL); (16) Puerto Rican in Puerto Rico (PUR); (17) Utah residents with Northern and Western European ancestry (CEU); (18) Finnish in Finland (FIN); (19) British in England and Scotland (GBR); (20) Iberian populations in Spain (IBS); (21) Toscani in Italy (TSI); (22) Bengali in Bangladesh (BEB); (23) Gujarati Indian in Houston, Texas (GIH); (24) Indian Telugu in the UK (ITU); (25) Punjabi in Lahore, Pakistan (PJL) and (26) Sri Lankan Tamil in the UK (STU).

### Structure analysis and Fst analysis

The Structure 2.3.4 software was used to analyze the structure of 27 populations, and Arlequin3.1 software was used to evaluate pairwise Fst values for assessing the relationship between 27 population groups. In addition, MEGA11 software was utilized to plot phylogenetic tree.

### Protein hazard prediction

We performed a functional analysis of missense variants using online tools such as Polyphen2 (http://genetics.bwh.harvard.edu/pph2/), SNAP2 (https://rostlab.org/services/snap/), Mutationassessor (http://mutationassessor.org/r3/), FATHMM (http://fathmm.biocompute.org.uk/index.html), and Mutationtaster (https://www.mutationtaster.org/) to assess the impact of SNVs mutations to predict protein function.

### Mutant protein structure prediction

A single amino acid change has the potential to significantly affect protein activity and function. We downloaded the protein structures of *CYP2D6* and *KCNH2* from the PDB database (https://www.rcsb.org/) and utilized the Chimera v1.16 software to predict and visualize the mutant protein structures.

### Statistical analysis

The data were compiled, ordered, and analyzed using Microsoft Excel 2019 (Microsoft, Redmond, WA, USA) and SPSS 26.0 (SPSS, Chicago, IL, USA). The χ^2^ test was utilized to estimate the Hardy–Weinberg equilibrium (HWE) and compare the divergences in genotype frequencies of 48 VIP variants between the Zhuang population and the other 26 populations. All statistical tests were two-tailed (*p* < 0.05). Bonferroni corrections were performed to determine the significance level. After the Bonferroni’s multiple tests, *p* < 4.01 × 10^−5^ was recognized as statistically significant.

### Ethics approval

This study was conducted by the World Medical Association Declaration of Helsinki and was approved by the Northwestern University Clinical Research Ethics Committee (Approval number of Ethics Committee: 230,413,002). All subjects signed an informed consent form.

## Results

### Basic characteristics of candidate VIP variants

The 48 VIP variants on 24 genes that satisfied the HWE equation (*p* > 0.05) were collected in this study. Table 1 summarizes the fundamental characteristics of these variants, including gene name, SNVs ID, position, functional consequence, genotype frequency, and minor allele frequency (MAF) in the Zhuang population. Additionally, Table S1 shows the PCR primers for the gathered VIP variants.

**Table 1:** Basic information of 48 selected VIP variants in the Zhuang population.

Genes	SNVs ID	Chr	BP	Functional consequence	Zhuang Allele		Genotype frequencies			MAF
					A	B	AA	AB	BB	
*CYP2J2*	rs11572325	1	59,896,030	Intron Variant	T	A	0 (0.000)	21 (0.105)	179 (0.895)	0.053
	rs10889160	1	59,896,449	Intron Variant	C	T	1 (0.005)	37 (0.185)	162 (0.810)	0.098
	rs890293	1	59,926,822	Upstream Transcript Variant	A	C	0 (0.000)	11 (0.055)	189 (0.945)	0.028
*DPYD*	rs1760217	1	97,137,438	Genic Downstream Transcript Variant, Intron Variant	G	A	11 (0.055)	80 (0.400)	109 (0.545)	0.255
	rs1801159	1	97,515,839	Coding Sequence Variant, Genic Downstream Transcript Variant, Intron Variant, Missense Variant	C	T	26 (0.131)	90 (0.455)	82 (0.414)	0.359
	rs1801265	1	97,883,329	Non-Coding Transcript Variant, Intron Variant, Coding Sequence Variant, 5 Prime UTR Variant, Missense Variant	G	A	0 (0.000)	40 (0.200)	160 (0.800)	0.100
*PTGS2*	rs5275	1	186,673,926	3 Prime UTR Variant	G	A	9 (0.046)	67 (0.340	121 (0.614)	0.216
*CACNA1S*	rs12139527	1	201,040,054	Missense Variant, Coding Sequence Variant, Intron Variant	G	A	2 (0.010)	36 (0.182)	160 (0.808)	0.101
	rs3850625	1	201,047,168	Coding Sequence Variant, Missense Variant	A	G	1 (0.005)	7 (0.035)	192 (0.960)	0.023
*RYR2*	rs2306238	1	237,550,803	Intron Variant	A	G	12 (0.060)	62 (0.312)	125 (0.628)	0.216
*ABCG2*	rs2231142	4	88,131,171	Coding Sequence Variant, Missense Variant	T	G	8 (0.040)	65 (0.327)	126 (0.633)	0.204
	rs2231137	4	88,139,962	Coding Sequence Variant, Missense Variant	T	C	27 (0.136)	99 (0.497)	73 (0.367)	0.384
*ADH1C*	rs698	4	99,339,632	Coding Sequence Variant, Non-Coding Transcript Variant, Missense Variant	C	T	2 (0.010)	49 (0.245)	149 (0.745)	0.133
*CYP3A5*	rs776746	7	99,672,916	Intron Variant, splice acceptor variant, genic Downstream Transcript Variant, Downstream Transcript Variant	T	C	19 (0.095)	1 (0.005)	180 (0.900)	0.098
*CYP3A4*	rs2242480	7	99,763,843	Intron Variant	T	C	16 (0.08)	83 (0.415)	101 (0.505)	0.288
*NAT2*	rs4646244	8	18,390,208	Upstream Transcript Variant, Genic Upstream Transcript Variant, Intron Variant	A	T	7 (0.035)	66 (0.330)	127 (0.635)	0.200
	rs4271002	8	18,390,758	Upstream Transcript Variant, Genic Upstream Transcript Variant, Intron Variant	C	G	4 (0.020)	50 (0.253)	144 (0.727)	0.146
	rs1041983	8	18,400,285	Coding Sequence Variant, Synonymous Variant	T	C	24 (0.120)	96 (0.480)	80 (0.400)	0.360
	rs1801280	8	18,400,344	Missense Variant, Coding Sequence Variant	C	T	1 (0.005)	6 (0.030)	193 (0.965)	0.020
	rs1799929	8	18,400,484	Coding Sequence Variant, Synonymous Variant	T	C	1 (0.005)	7 (0.035)	192 (0.960)	0.023
	rs1799930	8	18,400,593	Missense Variant, Coding Sequence Variant	A	G	7 (0.035)	69 (0.347)	123 (0.618)	0.209
	rs1208	8	18,400,806	Missense Variant, Coding Sequence Variant	G	A	1 (0.005)	7 (0.035)	192 (0.960)	0.023
	rs1799931	8	18,400,860	Missense Variant, Coding Sequence Variant	A	G	4 (0.020)	50 (0.250)	146 (0.730)	0.145
	rs1495741	8	18,415,371	None	A	G	28 (0.146)	90 (0.469)	74 (0.385)	0.380
*ALOX5*	rs2115819	10	45,405,641	Intron Variant	A	G	8 (0.040)	36 (0.181)	155 (0.779)	0.131
*CYP2C19*	rs12248560	10	94,761,900	Upstream Transcript Variant	T	C	0 (0.000)	1 (0.005)	199 (0.995)	0.003
	rs4244285	10	94,781,859	Coding Sequence Variant, Synonymous Variant	A	G	17 (0.085)	85 (0.425)	98 (0.490)	0.298
*CYP2C8*	rs7909236	10	95,069,673	Upstream Transcript Variant	T	G	2 (0.010)	44 (0.220)	154 (0.770)	0.120
	rs17110453	10	95,069,772	Upstream Transcript Variant	C	A	11 (0.055)	82 (0.410)	107 (0.535)	0.260
*CYP2E1*	rs3813867	10	133,526,101	Non-Coding Transcript Variant, Upstream Transcript Variant	C	G	4 (0.020)	49 (0.245)	147 (0.735)	0.143
	rs6413432	10	133,535,040	Intron Variant	A	T	0 (0.000)	44 (0.229)	148 (0.771)	0.115
	rs2070676	10	133,537,633	Intron Variant	G	C	10 (0.050)	57 (0.285)	133 (0.665)	0.193
*KCNJ11*	rs5219	11	17,388,025	Missense Variant, Stop Gained, 5 Prime UTR Variant, Intron Variant, Coding Sequence Variant	T	C	12 (0.061)	123 (0.628)	61 (0.311)	0.375
*SLCO1B1*	rs2306283	12	21,176,804	Missense Variant, Coding Sequence Variant	A	G	21 (0.106)	71 (0.357)	107 (0.538)	0.284
*CYP1A2*	rs762551	15	74,749,576	Intron Variant	C	A	12 (0.060)	90 (0.450)	98 (0.490)	0.285
	rs2472304	15	74,751,897	Intron Variant	A	G	2 (0.010)	43 (0.216)	154 (0.774)	0.118
*SULT1A1*	rs750155	16	28,609,251	5 Prime UTR Variant, Intron Variant, Genic Upstream Transcript Variant, Upstream Transcript Variant	C	T	28 (0.144)	118 (0.608)	48 (0.247)	0.448
*ACE*	rs1800764	17	63,473,168	None	C	T	23 (0.116)	102 (0.515)	73 (0.369)	0.374
	rs4291	17	63,476,833	Upstream Transcript Variant	T	A	0 (0.000)	177 (0.898)	20 (0.102)	0.449
	rs4267385	17	63,506,395	None	T	C	14 (0.070)	71 (0.357)	114 (0.573)	0.249
*CYP4F2*	rs2108622	19	15,879,621	Missense Variant, Coding Sequence Variant	T	C	3 (0.015)	66 (0.332)	130 (0.653)	0.181
	rs3093105	19	15,897,578	Missense Variant, Coding Sequence Variant	C	A	0 (0.000)	200 (1.000)	0 (0.000)	0.500
*CYP2A6*	rs8192726	19	40,848,591	Intron Variant	A	C	7 (0.035)	63 (0.315)	130 (0.650)	0.193
*SLC19A1*	rs1051298	21	45,514,912	Intron Variant, 3 Prime UTR Variant	G	A	34 (0.172)	120 (0.606)	44 (0.222)	0.475
	rs1051296	21	45,514,947	Intron Variant, 3 Prime UTR Variant	A	C	24 (0.122)	131 (0.668)	41 (0.209)	0.457
	rs1131596	21	45,538,002	Missense Variant, 5 Prime UTR Variant, Synonymous Variant, Genic Upstream Transcript Variant, Coding Sequence Variant	A	G	32 (0.162)	127 (0.644)	38 (0.193)	0.485
*CYP2D6*	rs1065852	22	42,130,692	Intron Variant, Missense Variant, Coding Sequence Variant	A	G	45 (0.238)	117 (0.619)	27 (0.143)	0.452
*KCNH2*	rs1805123	7	150,948,446	Missense Variant, Coding Sequence Variant, Genic Downstream Transcript Variant	G	T	151 (0.774)	44 (0.226)	0 (0.000)	0.113

SNVs: single nucleotide variants, Chr: chromosome, BP: base pairs, ID: identity documents, MAF: minor allele frequency.

### SNVs with significant differences in genotype frequencies between the Zhuang population and the other 26 populations

**Figure 1:**
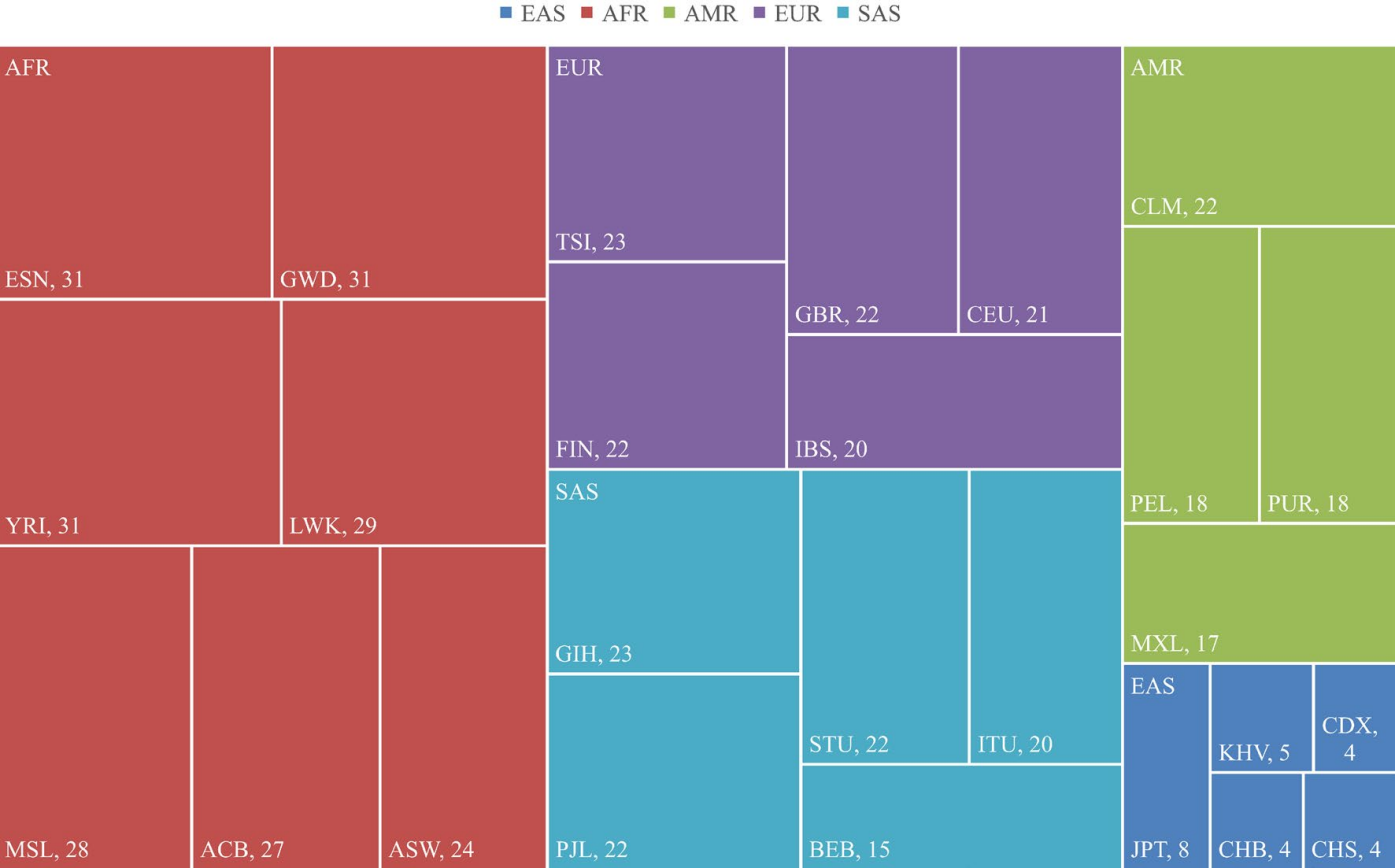
The amount of difference variants between the Zhuang population and 26 populations. The size of the rectangle indicates the number of different variants between the Zhuang population and the other 26 populations from five regions.

We compared the discrepancies in the genotype frequency distribution of the selected VIP variants between the Zhuang population and 26 other populations based on Chi-square tests. After the Bonferroni correction, the results were considered significant when *p* < 4.01 × 10^–5^. The number of SNVs with significant differences in genotype frequencies between the Zhuang population and 26 populations is shown in Fig. 1. The investigation demonstrated that the Zhuang population exhibited significant differences in four SNVs when compared to CDX, CHS, and KHV, and 31 SNVs when compared to ESN, GWD, and YRI. The Zhuang population exhibited differences in a number of SNVs when compared to other populations, including JPT (8), KHV (5), ACB (27), ASN (24), LWK (29), MSL (28), CLM (22), MXL (17), PEL (18), PUR (18), CEU (21), FIN (22), GBR (22), IBS (20), TSI (23), BEB (15), GIH (23), ITH (20), PJL (22), and STU (22). Furthermore, the Zhuang population showed significant differences in rs776746 (*CYP3A5*), rs4291 (*ACE*), and rs1805123 (*KCNH2*) compared to 26 other populations. Moreover, the Zhuang population exhibited significant differences in rs1065852 (*CYP2D6*) compared to 21 other populations (refer to Table 2 and Table S2).

**Table 2:** Genotype frequency distribution differences of 26 populations compared with the Zhuang population after Bonferroni’s multiple adjustment.

SNVs ID	Genes	EAS						AFR						AMR
		CDX	CHB	CHS	JPT	KHV	ACB	ASW	ESN	GWD	LWK	MSL	YRI	CLM
rs11572325	*CYP2J2*	–	0.376	–	–	–	**8.15E-06**	**3.82E-05**	**1.96E-05**	5.08E-05	1.51E-04	0.001	4.14E-05	0.024
rs10889160	*CYP2J2*	0.602	0.014	0.048	2.54E-04	0.201	**1.47E-19**	**9.71E-11**	**2.94E-22**	**8.02E-16**	**8.45E-17**	**1.82E-19**	**9.11E-22**	0.304
rs890293	*CYP2J2*	–	0.338	–	–	–	0.001	**1.17E-06**	**8.44E-10**	**1.99E-07**	**1.56E-06**	**2.57E-11**	**3.43E-07**	-
rs1760217	*DPYD*	0.054	0.391	0.306	0.002	0.978	0.091	0.697	0.612	0.007	0.093	0.436	0.020	0.037
rs1801159	*DPYD*	0.147	0.066	0.001	0.038	0.191	**2.47E-07**	6.90E-05	8.52E-05	**7.27E-13**	0.360	**2.49E-11**	**4.31E-07**	1.49E-04
rs1801265	*DPYD*	0.012	–	–	0.028	–	**1.64E-14**	**7.54E-19**	**1.56E-18**	**1.82E-21**	**3.06E-22**	**1.85E-14**	**1.34E-19**	**2.30E-05**
rs5275	*PTGS2*	0.947	0.088	0.452	0.546	0.780	**9.28E-19**	**2.50E-12**	**6.19E-23**	**5.67E-18**	**6.59E-19**	**1.92E-21**	**3.04E-23**	5.31E-05
rs12139527	*CACNA1S*	0.998	0.416	0.928	0.723	0.509	**1.10E-23**	**1.42E-19**	**1.26E-31**	**3.10E-34**	**1.04E-26**	**1.08E-30**	**7.57E-31**	0.451
rs3850625	*CACNA1S*	0.417	0.004	0.353	0.762	0.103	0.774	0.503	0.131	0.098	0.131	0.174	0.109	**1.52E-05**
rs2306238	*RYR2*	0.160	0.253	0.212	0.128	0.634	**1.70E-05**	0.004	**6.93E-08**	**1.02E-06**	**4.01E-06**	**4.26E-07**	**2.66E-06**	0.133
rs2231142	*ABCG2*	0.784	0.014	0.284	0.003	0.001	**1.30E-09**	0.004	**3.59E-11**	**1.42E-10**	**3.59E-11**	**5.40E-08**	**5.17E-12**	0.063
rs2231137	*ABCG2*	0.093	0.090	0.387	**2.13E-07**	0.907	**3.27E-17**	**1.40E-14**	**2.72E-17**	**2.11E-20**	**7.43E-11**	**6.58E-12**	**1.04E-17**	**2.06E-08**
rs698	*ADH1C*	0.577	0.005	0.043	0.048	0.146	0.336	0.409	0.044	0.201	0.946	0.361	0.048	0.001
rs776746	*CYP3A5*	**8.15E-22**	**5.32E-20**	**1.62E-19**	**5.81E-22**	**6.45E-22**	**2.28E-43**	**5.81E-38**	**9.74E-50**	**1.68E-46**	**7.50E-47**	**2.72E-45**	**2.35E-51**	**1.03E-13**
rs2242480	*CYP3A4*	0.715	0.415	0.420	0.540	0.660	**9.64E-26**	**1.65E-17**	**4.74E-38**	**7.33E-31**	**2.10E-38**	**1.13E-34**	**9.72E-33**	0.978
rs4646244	*NAT2*	0.456	0.936	0.162	0.078	2.04E-04	0.214	0.243	0.159	0.909	0.480	0.215	0.991	0.279
rs4271002	*NAT2*	2.87E-04	0.134	0.116	0.153	0.351	0.253	0.649	9.08E-05	2.06E-04	0.091	0.316	0.072	0.185
rs1041983	*NAT2*	0.002	0.988	0.068	0.248	7.78E-05	0.005	0.148	1.35E-04	0.613	0.206	0.001	0.003	0.220
rs1801280	*NAT2*	0.044	0.382	0.146	0.715	0.065	**4.85E-19**	**4.86E-19**	**1.62E-19**	**2.96E-24**	**1.05E-27**	**5.47E-16**	**5.09E-16**	**9.78E-27**
rs1799929	*NAT2*	0.077	0.498	0.224	0.762	0.201	**6.55E-15**	**6.25E-15**	**6.18E-13**	**3.38E-20**	**1.00E-23**	**9.37E-12**	**3.67E-09**	**1.12E-24**
rs1799930	*NAT2*	0.756	0.655	0.441	0.163	3.97E-04	0.339	0.141	0.200	0.499	0.197	0.381	0.923	0.338
rs1208	*NAT2*	0.077	0.498	0.132	0.762	0.058	**2.31E-25**	**7.37E-22**	**4.83E-27**	**9.05E-33**	**1.32E-32**	**4.64E-24**	**2.36E-27**	**1.05E-26**
rs1799931	*NAT2*	0.001	0.137	0.326	0.098	0.411	3.98E-04	0.018	**4.30E-06**	**1.84E-06**	**1.26E-06**	0.004	1.98E-04	0.024
rs1495741	*NAT2*	2.03E-04	0.908	0.014	0.509	**7.39E-06**	**1.42E-05**	**1.05E-07**	2.29E-04	5.63E-05	**3.17E-09**	0.002	0.007	**3.46E-12**
rs2115819	*ALOX5*	0.002	1.09E-04	0.040	0.003	0.051	**1.49E-38**	**8.51E-26**	**4.34E-38**	**4.74E-42**	**7.49E-34**	**9.70E-33**	**6.76E-41**	**4.00E-17**
rs12248560	*CYP2C19*	–	–	–	–	–	**4.50E-25**	**1.48E-17**	**7.51E-22**	**1.18E-21**	**1.46E-15**	**7.69E-23**	**4.81E-21**	**2.28E-11**
rs4244285	*CYP2C19*	0.606	0.197	0.100	0.800	0.905	0.001	0.002	0.054	**1.47E-05**	0.077	0.010	0.001	**2.61E-06**
rs7909236	*CYP2C8*	0.620	0.769	0.102	0.158	0.011	0.004	0.454	**1.43E-06**	**2.42E-07**	6.42E-05	**8.66E-06**	**4.56E-07**	**1.26E-06**
rs17110453	*CYP2C8*	0.927	0.100	0.026	0.005	0.575	**1.75E-13**	**1.36E-09**	**7.43E-14**	**5.84E-17**	**3.10E-15**	**1.82E-13**	**1.21E-15**	**8.33E-06**
rs3813867	*CYP2E1*	0.779	0.003	0.140	0.282	0.067	0.007	0.131	0.081	0.056	5.46E-05	0.023	0.097	0.820
rs6413432	*CYP2E1*	**1.81E-06**	**3.90E-07**	**7.62E-06**	**7.17E-06**	**2.88E-07**	–	–	0.119	0.031	–	0.020	0.237	0.039
rs2070676	*CYP2E1*	0.021	0.266	0.677	0.423	0.058	**1.78E-25**	**1.45E-13**	**1.04E-24**	**3.05E-26**	**8.00E-31**	**2.96E-26**	**3.50E-24**	0.073
rs5219	*KCNJ11*	1.13E-04	0.001	0.230	0.013	0.034	**2.98E-19**	**6.49E-09**	**5.01E-28**	**3.94E-29**	**3.34E-27**	**3.40E-25**	**8.78E-30**	**8.52E-07**
rs2306283	*SLCO1B1*	0.082	0.287	0.086	0.207	0.153	0.086	0.650	6.16E-05	0.036	0.004	0.082	0.023	**1.48E-06**
rs762551	*CYP1A2*	0.302	0.041	0.136	0.006	0.832	0.013	0.219	**3.48E-06**	0.001	**4.53E-08**	0.002	**2.86E-05**	0.321
rs2472304	*CYP1A2*	0.380	0.969	0.170	0.008	0.187	0.205	0.568	**7.35E-06**	4.86E-05	8.28E-05	4.17E-05	**8.77E-06**	**3.48E-12**
rs750155	*SULT1A1*	0.385	5.03E-05	0.596	**3.47E-06**	0.075	0.002	1.01E-04	**8.71E-10**	**9.53E-13**	**8.18E-06**	**1.42E-06**	**6.52E-08**	0.072
rs1800764	*ACE*	0.587	0.085	0.573	0.175	0.068	**1.07E-26**	**4.95E-17**	**4.17E-34**	**6.98E-40**	**4.78E-29**	**5.98E-37**	**2.54E-39**	0.080
rs4291	*ACE*	**2.44E-17**	**9.38E-22**	**7.87E-18**	**4.20E-15**	**1.50E-21**	**5.93E-16**	**4.76E-15**	**4.06E-17**	**1.65E-14**	**3.91E-21**	**1.36E-13**	**4.08E-22**	**2.53E-16**
rs4267385	*ACE*	0.548	0.987	0.413	0.580	0.412	**5.38E-26**	**1.54E-16**	**1.35E-27**	**1.57E-33**	**7.19E-35**	**1.15E-31**	**1.41E-31**	**9.42E-07**
rs2108622	*CYP4F2*	0.623	0.352	0.696	0.003	0.042	0.010	0.041	**4.11E-06**	1.84E-04	0.058	0.047	**1.65E-05**	0.004
rs3093105	*CYP4F2*	–	–	–	**2.56E-59**	–	**1.15E-37**	**1.86E-30**	**5.69E-33**	**2.44E-41**	**7.01E-38**	**2.46E-36**	**3.69E-35**	**5.66E-44**
rs8192726	*CYP2A6*	0.464	0.225	0.044	0.115	0.061	1.18E-04	0.043	0.010	**3.55E-05**	0.006	0.001	0.005	**3.04E-06**
rs1051298	*SLC19A1*	0.075	0.073	0.398	0.065	0.035	0.004	0.187	0.573	1.26E-04	0.046	0.054	0.149	0.097
rs1051296	*SLC19A1*	0.011	0.001	0.275	0.004	0.011	0.050	0.003	0.001	0.002	1.82E-04	0.034	0.011	0.003
rs1131596	*SLC19A1*	0.049	0.017	0.756	0.013	0.289	**3.32E-09**	0.024	0.002	**3.17E-14**	**1.37E-10**	**3.85E-09**	**2.32E-08**	0.022
rs1065852	*CYP2D6*	0.001	0.030	0.001	**5.47E-07**	0.005	**7.31E-23**	**4.59E-18**	**8.94E-30**	**1.34E-28**	**8.17E-37**	**3.59E-20**	**9.51E-29**	**1.66E-19**
rs1805123	*KCNH2*	**1.30E-51**	**3.59E-60**	**4.17E-60**	**3.57E-59**	**3.69E-53**	**4.89E-62**	**6.05E-51**	**1.44E-64**	**1.08E-66**	**1.29E-63**	**1.58E-61**	**1.60E-66**	**1.41E-42**

SNVs ID	Genes	AMR			EUR					SAS				
		MXL	PEL	PUR	CEU	FIN	GBR	IBS	TSI	BEB	GIH	ITU	PJL	STU
rs11572325	*CYP2J2*	–	–	7.89E-05	0.039	4.74E-04	0.158	0.044	0.049	–	–	0.372	–	–
rs10889160	*CYP2J2*	0.458	0.407	1.48E-04	0.382	**1.34E-05**	0.341	0.016	0.179	0.785	0.157	0.203	0.859	0.221
rs890293	*CYP2J2*	–	–	0.099	–	0.045	0.252	–	–	–	–	0.138	–	–
rs1760217	*DPYD*	0.819	0.206	0.002	0.862	0.008	0.013	0.026	0.066	0.184	0.671	**1.07E-05**	0.004	4.81E-05
rs1801159	*DPYD*	0.020	0.044	2.76E-04	**3.12E-06**	**2.63E-07**	**3.21E-05**	0.001	0.009	**2.43E-09**	**4.13E-10**	**4.57E-15**	**1.80E-10**	**4.52E-13**
rs1801265	*DPYD*	**9.52E-06**	0.013	**1.00E-06**	0.024	**2.85E-09**	0.008	1.50E-04	6.20E-05	0.003	**1.16E-11**	**4.66E-10**	**6.11E-09**	0.001
rs5275	*PTGS2*	0.005	**3.84E-05**	0.017	8.01E-05	0.457	0.099	0.007	0.053	7.24E-05	1.07E-04	0.001	**5.02E-09**	**1.52E-05**
rs12139527	*CACNA1S*	0.021	0.170	0.017	0.752	0.916	0.626	0.879	0.877	0.030	0.006	0.575	0.337	0.355
rs3850625	*CACNA1S*	1.91E-04	6.07E-05	0.127	0.001	**3.04E-11**	**1.07E-07**	0.001	**3.77E-05**	**2.24E-08**	**1.17E-19**	**2.68E-12**	**4.27E-11**	**7.10E-09**
rs2306238	*RYR2*	0.004	0.001	0.021	0.531	0.301	0.794	0.827	0.499	0.154	0.358	0.012	0.006	0.003
rs2231142	*ABCG2*	0.958	0.280	0.010	0.030	0.002	0.155	9.27E-05	**1.28E-05**	0.066	8.87E-05	0.010	0.009	0.001
rs2231137	*ABCG2*	0.001	0.237	**1.38E-08**	**3.39E-19**	**4.61E-13**	**2.17E-18**	**4.02E-18**	**5.28E-15**	0.002	**8.91E-09**	**1.42E-12**	**1.07E-11**	**1.12E-05**
rs698	*ADH1C*	2.91E-04	0.354	**2.54E-10**	**1.08E-17**	**5.94E-19**	**6.67E-14**	**9.12E-07**	**9.77E-07**	0.302	**2.55E-05**	0.002	**1.37E-07**	**2.15E-08**
rs776746	*CYP3A5*	**1.45E-17**	**1.19E-09**	**1.15E-15**	**1.61E-05**	**7.01E-06**	**1.47E-06**	**2.30E-07**	**1.23E-06**	**5.72E-24**	**5.31E-21**	**2.07E-22**	**1.10E-21**	**2.70E-22**
rs2242480	*CYP3A4*	0.068	**1.40E-09**	0.137	**5.05E-10**	**1.86E-08**	**2.29E-08**	**4.78E-06**	**5.34E-08**	0.088	0.479	0.060	0.010	0.130
rs4646244	*NAT2*	0.122	0.004	0.801	0.003	0.067	0.018	0.042	0.072	0.210	**2.12E-05**	4.70E-04	4.15E-04	**9.95E-07**
rs4271002	*NAT2*	0.535	0.015	0.200	0.027	0.584	0.779	0.775	0.647	0.241	0.930	0.415	0.909	0.788
rs1041983	*NAT2*	0.185	0.005	0.184	0.072	0.224	0.098	0.480	0.269	0.870	0.112	0.289	0.201	0.003
rs1801280	*NAT2*	**4.50E-24**	**1.34E-20**	**1.78E-27**	**3.56E-31**	**2.53E-33**	**5.89E-33**	**1.73E-36**	**2.44E-32**	**1.69E-24**	**5.68E-24**	**1.80E-25**	**7.16E-31**	**1.41E-20**
rs1799929	*NAT2*	**1.84E-22**	**3.15E-19**	**1.08E-23**	**2.04E-30**	**3.87E-31**	**1.07E-30**	**1.03E-35**	**1.30E-31**	**2.11E-22**	**7.73E-21**	**7.06E-23**	**1.13E-26**	**9.82E-19**
rs1799930	*NAT2*	0.148	0.001	0.782	0.007	0.233	0.019	0.031	0.097	0.263	**1.11E-05**	0.001	2.15E-04	**2.54E-07**
rs1208	*NAT2*	**6.69E-28**	**7.16E-20**	**9.13E-27**	**4.93E-29**	**1.85E-30**	**1.02E-30**	**9.31E-36**	**2.80E-32**	**1.17E-26**	**2.75E-23**	**3.12E-24**	**7.53E-31**	**2.92E-22**
rs1799931	*NAT2*	0.479	0.030	0.073	**3.37E-07**	2.68E-04	4.28E-05	9.27E-05	**4.32E-06**	0.242	0.004	0.007	0.008	0.055
rs1495741	*NAT2*	**1.12E-07**	0.004	**7.38E-11**	**1.93E-13**	**1.66E-15**	**8.63E-16**	**6.88E-20**	**6.96E-14**	**1.18E-12**	**1.13E-17**	**3.28E-15**	**6.09E-21**	**2.66E-19**
rs2115819	*ALOX5*	**1.53E-11**	**2.59E-07**	**1.26E-15**	**4.32E-25**	**3.56E-20**	**3.24E-19**	**6.08E-21**	**7.28E-22**	**9.82E-17**	**4.99E-24**	**1.42E-21**	**1.00E-16**	**2.89E-15**
rs12248560	*CYP2C19*	**5.71E-09**	-	**7.65E-16**	**1.15E-20**	**1.68E-19**	**2.79E-21**	**1.33E-19**	**1.33E-19**	-	**7.28E-12**	**5.54E-12**	**3.36E-12**	**1.76E-11**
rs4244285	*CYP2C19*	4.23E-04	**2.99E-09**	**2.39E-05**	4.15E-05	0.058	3.32E-04	9.91E-05	**6.12E-08**	0.702	0.235	0.157	0.095	0.018
rs7909236	*CYP2C8*	**1.27E-05**	**8.08E-10**	0.070	4.53E-05	**3.35E-05**	0.006	0.071	0.132	0.010	0.001	0.010	0.016	0.227
rs17110453	*CYP2C8*	0.001	**1.36E-08**	6.18E-05	**2.48E-06**	0.273	**3.68E-05**	0.189	**9.12E-06**	0.105	0.094	0.158	0.369	0.017
rs3813867	*CYP2E1*	0.832	0.780	0.013	0.013	3.79E-04	1.63E-04	**1.83E-05**	0.003	**1.20E-05**	**1.00E-06**	**1.16E-06**	**2.78E-06**	**3.11E-07**
rs6413432	*CYP2E1*	0.114	0.008	0.015	0.361	0.015	0.060	0.078	0.335	0.002	**3.46E-06**	0.003	0.106	0.002
rs2070676	*CYP2E1*	0.299	0.113	0.020	0.074	0.005	0.024	0.253	0.051	0.578	0.322	0.705	0.492	0.886
rs5219	*KCNJ11*	0.103	0.106	3.07E-04	0.384	0.037	0.004	0.018	0.001	0.002	0.001	0.013	**2.17E-06**	0.005
rs2306283	*SLCO1B1*	**7.78E-10**	**3.53E-07**	1.09E-04	**8.99E-11**	**5.18E-09**	**5.74E-13**	**2.70E-11**	**1.20E-13**	0.004	0.001	0.022	**9.27E-08**	0.001
rs762551	*CYP1A2*	0.029	2.17E-04	0.842	0.503	0.255	0.321	0.054	0.022	0.001	**5.13E-06**	**2.96E-05**	5.70E-05	**1.26E-07**
rs2472304	*CYP1A2*	1.01E-04	0.989	**2.22E-19**	**1.27E-31**	**3.26E-24**	**3.18E-30**	**2.90E-28**	**7.29E-22**	0.084	0.234	0.790	0.001	0.921
rs750155	*SULT1A1*	0.218	**1.17E-12**	6.26E-05	0.004	0.330	0.005	0.002	3.76E-04	**3.83E-12**	**6.89E-08**	**6.20E-11**	**2.10E-08**	**1.25E-17**
rs1800764	*ACE*	0.129	0.001	0.079	0.011	0.172	0.114	0.384	0.001	0.685	0.147	0.210	0.580	0.949
rs4291	*ACE*	**3.27E-19**	**2.23E-26**	**9.16E-16**	**1.28E-17**	**1.51E-12**	**2.50E-18**	**1.41E-15**	**8.52E-19**	**3.55E-16**	**6.16E-16**	**5.81E-22**	**5.34E-17**	**6.88E-16**
rs4267385	*ACE*	3.89E-04	0.875	**4.81E-10**	**3.57E-11**	**2.23E-10**	**1.49E-12**	**1.72E-13**	**3.89E-22**	0.074	4.20E-04	0.131	2.91E-04	0.004
rs2108622	*CYP4F2*	0.188	0.123	0.002	0.016	0.345	0.001	**3.00E-06**	**3.05E-06**	**8.50E-09**	**7.07E-11**	**6.54E-09**	**5.88E-08**	**5.84E-10**
rs3093105	*CYP4F2*	**1.39E-42**	**1.35E-55**	**1.54E-44**	**1.21E-44**	**1.84E-49**	–	**7.51E-35**	**1.34E-39**	**6.52E-45**	**1.47E-46**	**1.85E-42**	**3.34E-42**	**2.80E-46**
rs8192726	*CYP2A6*	3.72E-04	4.46E-05	**3.48E-06**	**2.15E-05**	0.090	**6.09E-06**	**1.27E-05**	1.38E-04	0.096	0.213	0.019	0.356	0.019
rs1051298	*SLC19A1*	**2.48E-05**	1.15E-04	0.092	0.005	0.083	3.25E-04	0.008	0.023	0.196	0.029	0.205	0.342	0.021
rs1051296	*SLC19A1*	**6.24E-08**	**9.47E-08**	0.001	7.75E-05	0.002	**1.09E-06**	1.71E-04	1.69E-04	0.092	0.025	0.002	0.003	0.003
rs1131596	*SLC19A1*	**4.37E-07**	**1.21E-05**	0.006	0.045	0.022	**2.49E-06**	0.001	0.003	0.002	0.001	4.88E-04	2.09E-04	0.001
rs1065852	*CYP2D6*	**6.20E-18**	**8.28E-30**	**1.30E-21**	**8.70E-14**	**4.41E-23**	**5.54E-14**	**4.52E-21**	**8.40E-18**	**1.55E-13**	**1.24E-22**	**1.76E-20**	**4.59E-27**	**3.85E-23**
rs1805123	*KCNH2*	**6.73E-41**	**3.21E-51**	**3.55E-41**	**3.82E-40**	**1.03E-44**	**4.00E-36**	**8.87E-38**	**9.24E-37**	**4.67E-34**	**3.55E-39**	**1.83E-37**	**3.46E-42**	**7.87E-42**

Bolded font indicates significant results.

EAS, East Asian; SAS, South Asian; EUR, European; AFR, African; AMR, American; CDX, Chinese Dai in Xishuangbanna, China; CHB, Han Chinese in Beijing, China; CHS, Southern Han Chinese, China; JPT, Japanese in Tokyo, Japan; KHV, Kinh in Ho Chi Minh City; Vietnam; BEB, Bengali in Bangladesh; GIH, Gujarati Indian in Houston, Texas; ITU, Indian Telugu in the UK; PJL, Punjabi in Lahore, Pakistan; STU, Sri Lankan Tamil in the UK; CEU, Western European ancestry; FIN, Finnish in Finland; GBR, British in England and Scotland; IBS, Iberian populations in Spain; TSI, Toscani in Italy; ACB, African Caribbeans in Barbados; ASW, African Ancestry in Southwest USA; ESN, Esan in Nigeria; GWD, Gambian in Western Divisions, The Gambia; LWK, Luhya in Webuye, Kenya; MSL, Mende in Sierra Leone; YRI, Yoruba in Ibadan, Nigeria; CLM, Colombian in Medellin, Colombia; MXL, Mexican Ancestry in Los Angeles, Colombia; PEL, Peruvian in Lima, Peru; PUR, Puerto Rican in Puerto Rico.

### Genetic structure analysis of 27 populations

**Figure 2:**
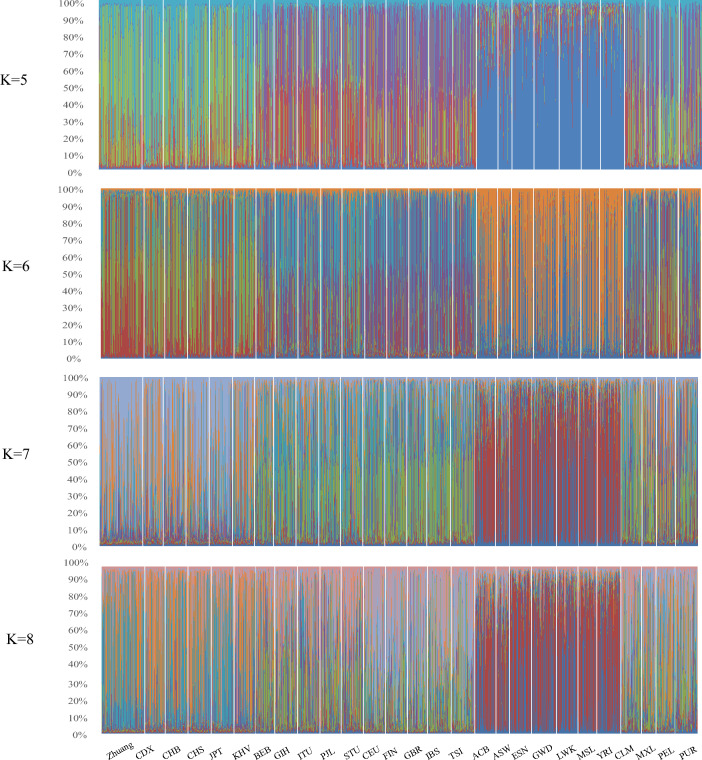
Structure analysis of the genetic relationship between the Zhuang population and the other 26 populations. K denotes the possible numbers of parental population clusters. Each vertical bar represents a sample, dividing into color sections. K = 5 were utilized to evaluate the relationship between Zhuang and 26 populations.

A model-based clustering approach was used to analyze the genetic structure of the 27 populations distributed in Africa, America, East Asia, Europe and South Asia to further analyze their relationship. Based on the Structure 2.3.1 Software, different K values ranging from 5 to 8 were hypothetically considered in structure analysis. When K = 5, the groups were divided into 5 subgroups based on the relative majority probability of assigning individuals to subgroups (subgroup 1: GWD and LWK; Subgroup 2: BEB, CEU, FIN, GBR, IBS, TSI, CLM, MXL and PUR; Subgroup 3: Zhuang, CDX, CHB, CHS, JPT, KHV and PUR; Subgroup 4: GIH, ITU, PJL and STU; Subgroup 5: ACB, ASW, ESN, MSL and YRI). It can be observed from Fig. 2 that Zhuang population have a stronger affinity with CDX, CHB, CHS, JPT, KHV and PUR. This is consistent with the results in Table 2.

**Figure 3:**
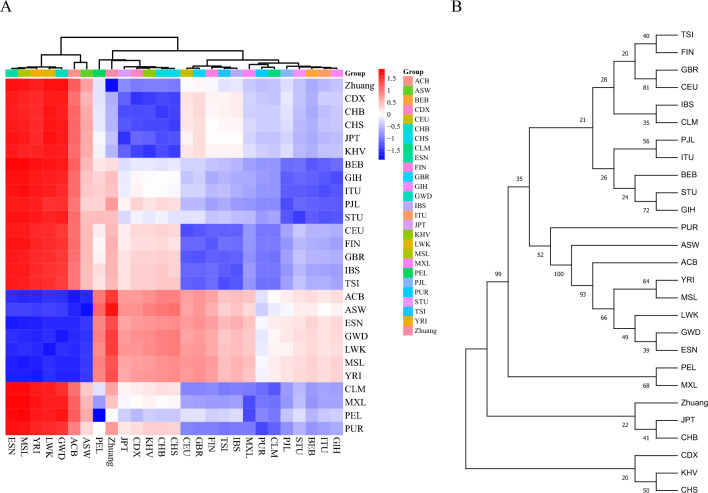
Fst value heamap and phylogenetic tree among 27 populations. (**A**) Heatmap based on the pairwise Fst values between 27 populations. (**B**) The phylogenetic tree was constructed by the neighboring-joining method among 27 populations.

The pairwise Fst values were used to assess relationships among 27 populations, as shown in Table 3 and Fig. 3A. The Fst values between the Zhuang population and the East Asian population (CDX, CHB, CHS, JPT and KHV) were small, which were 0.065, 0.068, 0.066, 0.073 and 0.067, respectively (Table 3). Smaller Fst values indicate closer relationships between the two groups and suggest that they share similar genetic backgrounds. The result is confirmed by the phylogenetic trees of 27 populations shown in Fig. 3B.

**Table 3:** Pairwise Fst values among the Zhuang and 26 populations.

	Zhuang	CDX	CHB	CHS	JPT	KHV	BEB	GIH	ITU	PJL	STU	CEU	FIN	GBR	IBS	TSI	ACB	ASW	ESN	GWD	LWK
Zhuang	0.000																				
CDX	0.065	0.000																			
CHB	0.068	0.013	0.000																		
CHS	0.066	0.009	0.005	0.000																	
JPT	0.073	0.026	0.012	0.015	0.000																
KHV	0.069	0.007	0.013	0.006	0.024	0.000															
BEB	0.092	0.067	0.064	0.064	0.060	0.066	0.000														
GIH	0.108	0.082	0.081	0.079	0.072	0.078	0.010	0.000													
ITU	0.103	0.082	0.076	0.075	0.069	0.078	0.006	0.005	0.000												
PJL	0.122	0.095	0.094	0.091	0.082	0.091	0.010	0.007	0.006	0.000											
STU	0.108	0.077	0.075	0.070	0.065	0.071	0.012	0.011	0.007	0.011	0.000										
CEU	0.140	0.123	0.124	0.122	0.111	0.122	0.057	0.054	0.058	0.047	0.069	0.000									
FIN	0.138	0.115	0.115	0.110	0.104	0.115	0.049	0.042	0.047	0.037	0.057	0.010	0.000								
GBR	0.146	0.128	0.131	0.128	0.119	0.128	0.057	0.056	0.059	0.047	0.069	0.005	0.010	0.000							
IBS	0.129	0.116	0.119	0.114	0.105	0.115	0.042	0.040	0.044	0.033	0.053	0.011	0.012	0.009	0.000						
TSI	0.125	0.114	0.117	0.116	0.105	0.115	0.048	0.045	0.050	0.041	0.059	0.011	0.015	0.008	0.006	0.000					
ACB	0.237	0.182	0.195	0.196	0.180	0.188	0.146	0.145	0.141	0.136	0.143	0.179	0.175	0.186	0.162	0.158	0.000				
ASW	0.198	0.147	0.158	0.161	0.146	0.154	0.107	0.107	0.102	0.097	0.108	0.138	0.134	0.144	0.125	0.121	0.009	0.000			
ESN	0.283	0.228	0.236	0.240	0.221	0.232	0.188	0.187	0.179	0.176	0.180	0.230	0.225	0.237	0.213	0.206	0.013	0.021	0.000		
GWD	0.280	0.226	0.235	0.239	0.219	0.231	0.179	0.178	0.170	0.168	0.176	0.213	0.208	0.219	0.194	0.187	0.011	0.019	0.013	0.000	
LWK	0.283	0.227	0.241	0.242	0.226	0.233	0.181	0.179	0.174	0.165	0.174	0.222	0.213	0.225	0.199	0.194	0.015	0.023	0.012	0.016	0.000
MSL	0.277	0.222	0.233	0.236	0.218	0.227	0.188	0.188	0.181	0.177	0.181	0.229	0.225	0.235	0.210	0.204	0.009	0.022	0.006	0.010	0.016
YRI	0.272	0.220	0.228	0.233	0.213	0.226	0.184	0.183	0.176	0.174	0.178	0.222	0.218	0.229	0.204	0.197	0.008	0.020	0.004	0.008	0.015
CLM	0.107	0.077	0.084	0.082	0.075	0.080	0.029	0.031	0.033	0.028	0.042	0.021	0.021	0.020	0.018	0.017	0.128	0.093	0.172	0.157	0.164
MXL	0.112	0.084	0.082	0.087	0.080	0.090	0.032	0.042	0.040	0.032	0.052	0.040	0.036	0.039	0.038	0.037	0.159	0.116	0.200	0.190	0.190
PEL	0.125	0.091	0.097	0.098	0.101	0.102	0.088	0.100	0.098	0.096	0.108	0.110	0.100	0.110	0.107	0.104	0.201	0.165	0.242	0.237	0.231
PUR	0.103	0.078	0.084	0.082	0.074	0.084	0.028	0.035	0.032	0.026	0.039	0.023	0.021	0.021	0.018	0.017	0.112	0.079	0.153	0.139	0.143

	LWK	MSL	YRI	CLM	MXL	PEL	PUR														
Zhuang																					
CDX																					
CHB																					
CHS																					
JPT																					
KHV																					
BEB																					
GIH																					
ITU																					
PJL																					
STU																					
CEU																					
FIN																					
GBR																					
IBS																					
TSI																					
ACB																					
ASW																					
ESN																					
GWD																					
LWK	0.000																				
MSL	0.016	0.000																			
YRI	0.015	0.004	0.000																		
CLM	0.164	0.171	0.165	0.000																	
MXL	0.190	0.201	0.194	0.017	0.000																
PEL	0.231	0.244	0.235	0.058	0.035	0.000															
PUR	0.143	0.151	0.146	0.009	0.019	0.067	0.000

### Genotype frequencies of four significantly different SNVs

**Figure 4:**
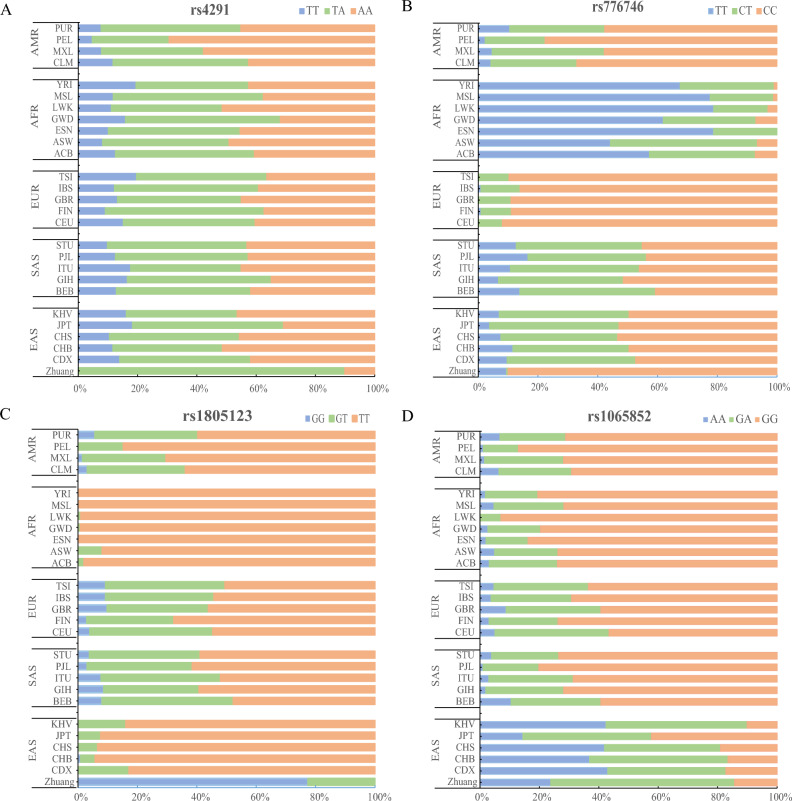
The distribution of genotype frequencies for significantly different SNVs in 27 populations at the rs776746, rs4291, rs1805123 and rs1065852.

Moreover, the genotype frequency distribution of rs776746 (*CYP3A5*), rs4291 (*ACE*), rs1805123 (*KCNH2*), and rs1065852 (*CYP2D6*) in 26 populations are shown in Fig. 4. The genotype frequency of rs4291-AT in the Zhuang population is remarkably higher than that of the other 26 populations. The CC genotype frequency of rs776746 is similar to that of EUR and significantly higher than that of AFR. In the Zhuang population, the frequency of the rs1805123-GG genotype is notably higher compared to that observed in the other 26 populations. The frequency of rs1065852-GG is similar to that of KHV, CHS, CHB, and CDX, and lower than that of other populations.

### MAF distribution of four significantly different SNVs

**Figure 5:**
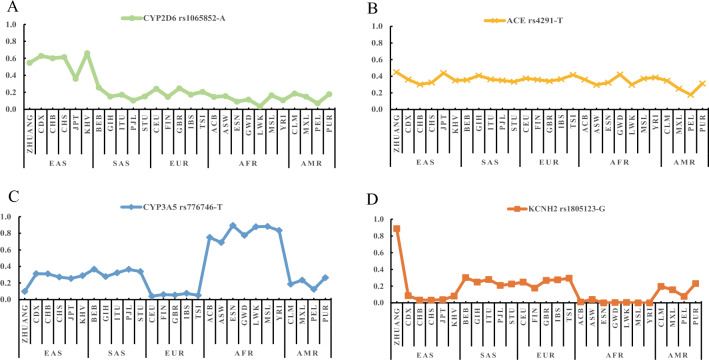
The map of the allele frequency distribution for significantly different SNVs in 27 populations at the rs1065852, rs4291, rs776746 and rs1805123.

Based on the allele frequencies calculated in this study, we plotted a map of the MAF distribution of VIP variants that substantially differed from the other 26 populations. According to Fig. 5, the allele frequency of rs776746 at *CYP3A5* in the Zhuang population was similar to that in the European population, despite their close genetic affinity with East Asians. The G allele of rs1805123 at *KCNH2* was nearly fixed in the Zhuang population and had a low frequency in other global populations. The MAF of rs1065852 (*CYP2D6*) was similar to that of East Asians and higher than other populations. However, there were no significant differences in T allele frequency for rs4291 at ACE among different populations.

### Clinical relevance of significant variants

The Table 4 presents the clinical annotation information of the VIP variants in PharmGKB. The genotype frequency of rs776746 (*CYP3A5*) has been shown to have an impact on the dose, toxicity and metabolism of tacrolimus^19–21^. Specific mutations in rs4291 (*ACE*) have been implicated in the metabolism of anti-hypertensive drugs such as amlodipine, sodium chlorthalidone and lisinopril^22^. Furthermore, they also influenced the risk of aspirin intolerance in asthmatics exposed to aspirin^23^. The efficacy of metoclopramide in patients with gastric disease was found to be associated with rs1805123 (*KCNH2*)^24^. Rs1065852 played an indispensable role in the regulation of the α-hydroxymetoprolol metabolism in patients with non-small cell lung cancer^25^.

**Table 4:** Clinical annotation of very important pharmacogenomic variants with significant differences.

Gene	Variant	PMID	Molecules	Association	*P*-value	Type	Phenotype
*CYP3A5*	rs776746	23,073,468	Tacrolimus	Genotype CC is associated with decreased dose of tacrolimus in people with Kidney Transplantation as compared to genotypes CT + TT	0.016	Dosage	Kidney Transplant
*CYP3A5*	rs776746	21,677,300	Tacrolimus	Allele T is associated with increased risk of tacrolimus nephrotoxicity when treated with tacrolimus in people with Kidney Transplantation as compared to allele C	0.025	Toxicity	Kidney Transplant
*CYP3A5*	rs776746	24,120,259	Tacrolimus	Genotype CT is associated with increased dose of tacrolimus in people with Kidney Transplantation as compared to genotype CC	< 0.001	Metabolism/PK	Kidney Transplant
*ACE*	rs4291	27,546,928	Captopril	Genotype AA is associated with decreased severity of Kidney Failure when treated with captopril in people with Alzheimer Disease as compared to genotypes AT + TT	0.029	Efficacy	Alzheimer disease
*ACE*	rs4291	18,727,619	Aspirin	Genotypes AT + TT are associated with increased risk of aspirin intolerance when exposed to aspirin in people with Asthma as compared to genotype AA	0.015	Toxicity/ADR	Asthma
*ACE*	rs4291	20,577,119	Amlodipine/lisinopril/chlorthalidone	Genotypes AA + AT are associated with decreased fasting glucose when treated with amlodipine, chlorthalidone or lisinopril in people with Hypertension as compared to genotype TT	0.001	Efficacy	Anti-Hypertension
*CYP2D6*	rs1065852	10,223,777	Alpha-hydroxymetoprolol	Allele A is associated with decreased clearance of alpha-hydroxymetoprolol in healthy individuals as compared to allele G	< 0.050	Metabolism/PK	Carcinoma, Non-Small-Cell LungMesothelioma
*CYP2D6*	rs1065852	24,528,284	Citalopramescitalopram	Allele A is associated with plasma concentration of S-didesmethyl-citalopram when treated with citalopram or escitalopram in people with Depressive Disorder, Major as compared to allele G	2E-16	Other	Depressive Disorder
*CYP2D6*	rs1065852	23,277,250	Iloperidone	Genotype GG is associated with increased QTc interval when treated with iloperidone in people with Schizophrenia as compared to genotypes AA + AG	0.028	Other	Schizophrenia
*KCNH2*	rs1805123	22,688,145	Metoclopramide	The efficacy of metoclopramide in patients with gastric disease was correlated with the polymorphism of *KCNH2* (rs1805123, *P* = 0.020) gene	0.020	Dose effect	Gastric disease

ADR: Adverse drug reactions.

### Prediction of functional damage in proteins

**Figure 6:**
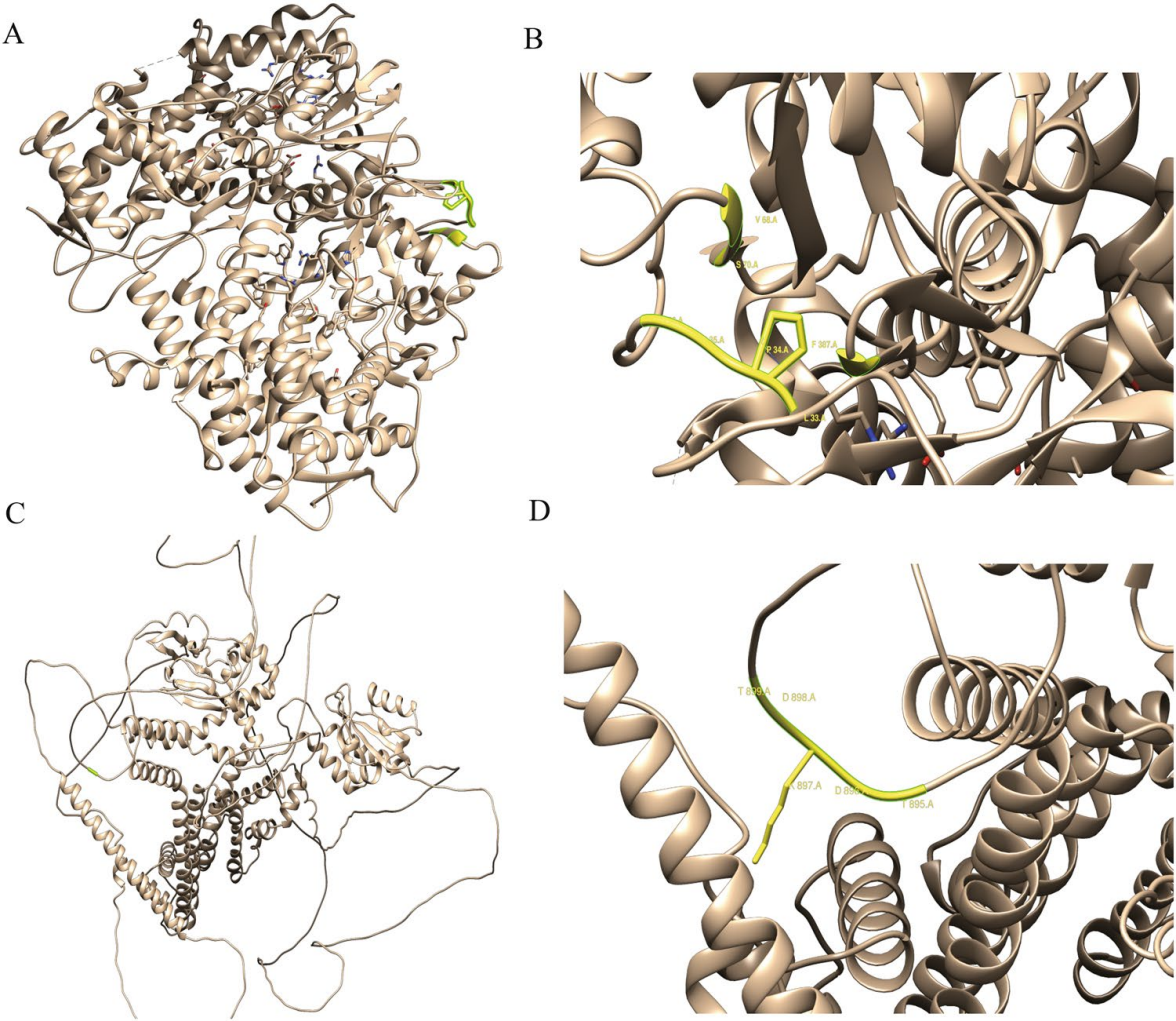
Structural prediction of point mutated proteins. (**A**) 3D structure of the *CYP2D6* protein, with the yellow part being a SNV. (**B**) rs1065852 mutated local structure. (**C**) 3D structure of the *KCNH2* protein, with the yellow part being a SNV mutation. (**D**) rs1805123 mutated local structure.

Subsequently, we used the PolyPhen-2, SNAP2, FATHMM, Mutationtaster, and Mutationassessor online databases to predicte whether the four SNVs would affect protein structure and function (Table 5). The results indicated that rs1805123 would cause a mutation from K to T at position 897 of *KCNH2*, however, this mutation was considered benign and less harmful to the protein in most databases. In contrast, rs1065852 caused a mutation from P to A at the 34th position of *CYP2D6*. The database predicted that this change would severely impair the protein's function and potentially contribute to certain diseases. Additionally, Chimera v1.16 was utilized for predicting the structure of point mutations of *CYP2D6* and *KCNH2*, as shown in Fig. 6.

**Table 5:** The functional analysis of missense variants using PolyPhen-2, SNAP2, Mutationassessor, FATHMM, and Mutationtaster.

SNVs ID	Gene	AA change	PolyPhen-2		SNAP2		FATHMM		Mutation taster		Mutationassessor	
			Score	Predicted effect	Score	Predicted effect	Coding Score	Predicted effect	Prob	Predicted	Func.Impact	FI score
rs1805123	*KCNH2*	K897T	0	Benign	-53	Neutral	0.760	pathogenic	0.205	polymorphism	low	1.735
rs1065852	*CYP2D6*	P34A	0.953	Deleterious	60	Effect	0.820	pathogenic	0.999	disease causing	high	4.080

## Discussion

During the development of biological sciences, it has gradually been realized that genetic differences between populations have an essential influence on drug metabolism, dosages and ADRs. This can potentially affect the efficacy of certain medications in specific populations. Pharmacogenomics research is gradually illuminating the genetic factors responsible for variations in drug utilization among diverse populations. For instance, an important study conducted by Wen et al. demonstrated that there were significant differences in allele frequencies of key genetic variants affecting drug selection and dosing between Hmong and East Asian populations^26^. Furthermore, pharmacogenomics studies have been reported on Mongolian^27^, Tibetan^28^, and Blang^29^, among others. However, there have been few pharmacogenomics studies conducted on the Zhuang population.

In this study, 200 Zhuang subjects in Yunnan Province were recruited and genotyped for 48 VIP variants on 24 candidate genes. The genotypic distribution was compared to that of 26 populations from the 1000G dataset. The results revealed significant differences in *CYP3A5* (rs776746), *ACE* (rs4291), *KCNH2* (rs1805123), and *CYP2D6* (rs1065852) between Zhuang population and the other 26 populations. We also used the PharmGKB database to annotate significantly different SNVs. Our study on VIP polymorphism in the Zhuang population may provide tailored therapy for the Zhuang population.

The cytochrome P450 (CYP) superfamily is an ancient enzyme family found in hundreds of eukaryotic and prokaryotic organisms^30^. The human genome encodes 57 putative functional CYP genes, as well as 58 pseudogenes. Among these 57 functional human CYPs, 12 are involved in the metabolism of 70–80% commonly used drugs, including *CYP2D6* and *CYP3A5*^31^. The human *CYP2D6* gene is relatively short, spanning only about 4.3 Kbps on the long arm of chromosome 22 (22q13.2). The CYP2D6 gene is composed of 9 exons and encodes the CYP2D6 protein, which is localized in the endoplasmic reticulum. This protein exhibited highly expressed levels in the liver, brain, intestinal tissues, and lymphocytes^32^. There were large population differences in the distribution of *CYP2D6* alleles, which could lead to variations in drug utilization among different populations^33^. rs1065852 has been reported to result in reduced protein stability and a poor response to drugs such as iloperidone, atorvastatin, antidepressants, and antipsychotics^34–37^. Moreover, allele A was associated with decreased clearance of alpha-hydroxymetoprolol in healthy individuals and a higher plasma concentration of S-didesmethyl-citalopram when treated with citalopram or escitalopram in people with Depressive Disorder compared to allele G^25,38^. In this study, the MAF of rs1065852-A (54.80%) was higher than that in SAS (16.70%), EUR (20.30%), AFR (11.60%), and AMR (14.60%). In addition, the frequency of rs1065852-GG was lower than that in other populations except for EAS. Therefore, the differences in drug efficacy and safety caused by *CYP2D6* rs1065852 should be taken into consideration in the Zhuang population.

*CYP3A5*, which is located in chromosome 7q21.1, is involved in the metabolism of many drugs. Tacrolimus, one of the substrates of *CYP3A5*, is widely used as an immunosuppressive agent for organ transplantation^39^. The expression of *CYP3A5* varied among different populations, which may have an impact on drug metabolism in those populations^21^. One study identified that genotype CT was associated with a higher tacrolimus dose in renal transplant patients compared to genotype CC^21^. The results of Flores-Pérez et al. revealed that critically ill Mexican pediatric patients with the CYP3A5*3 allele variant (rs776746) had increased plasma levels of midazolam and higher drug clearance 3 h after the end of the infusion compared to carriers with the normal allele^40^. The study by Liang et al. pointed out that individuals with the rs776746-CC had an increased risk of amlodipine-induced peripheral edema in a dominant model among Chinese Han hypertensive patients^41^. In our study, the frequencies of CT, TT and CC of rs776746 were 0.5%, 9.5% and 90.0%, separately. The frequency of CT genotype in the Zhuang was lower than that in the other 26 populations, highlighting the importance of considering metabolism and absorption of specific drugs in the Zhuang population.

*KCNH2* is a gene that encodes a component of voltage-activated potassium channel found in cardiac muscle, neuronal cells, and microglia. Four copies of this protein interact with a copy of the KCNE2 protein to form a functional potassium channel. Mutations in this gene can lead to long QT syndrome type 2 (LQT2)^42^. A recent study has identified *KCNH2* p.Gly262AlafsTer98 as a novel pathogenic variant associated with long QT syndrome in a Spanish population^43^. In a separate study, it was found that *KCNH2* mutations cause fetal biventricular densified cardiomyopathy with pulmonary stenosis and bradycardia^44^. The efficacy of metoclopramide in patients with gastric disease was found to be correlated with the polymorphism of *KCNH2* gene (rs1805123, *p* = 0.020)^24^. A Marjamaa et al. found that allele G of rs1805123 was associated with a shorter QT interval in a Finnish population compared to the TT genotype^45^. In our study, the frequency of rs1805123-G was significantly higher in the Zhuang population than in the other 26 populations (88.70%). The rs1805123 causes a K-T mutation at site 897 of *KCNH2*. Although most databases predict this mutation to be benign, attention should be paid to the shorter/long QT interval and the dose of metoclopramide in the Zhuang population.

*ACE*, which encodes an enzyme, is known to participate in the regulation of blood pressure and electrolyte balance. Numerous studies have shown that *ACE* is closely associated with nervous system diseases^46,47^, cardiovascular diseases^48^, and hypertension^49,50^. In a previously published study, we found that the rs4291 genotype influenced drug dosing in the treatment of the disease. De Oliveira et al. found a correlation between the use of brain-penetrating angiotensin converting enzyme inhibitors (ACEIs) (such as captopril or perindopril) in antihypertensive therapy and rs4291^46^. Another study has confirmed that the AA genotype of rs4291, compared to the genotype AT + TT, is associated with a reduced severity of renal failure in patients with Alzheimer's disease treated with captopril^51^. Furthermore, rs1800764 and rs4291 also formed haplotypes. A study discovered that ACEIs decelerated cognitive decline in individuals carrying the *ACE* haplotype with rs1800764-T and rs4291-A, as well as those carrying the *APOE4* haplotype with either rs1800764-T or rs4291-T, regardless of changes in blood pressure^52^. Our study demonstrated that the allele frequency of rs4291 (*ACE*) differed significantly between the Zhuang population and the other 26 populations, which has been found to be associated with drug metabolism for various diseases, such as captopril, aspirin, and amlodipine. It may provide guidance for precision drug administration in the Zhuang population.

Our results are likely to complement the pharmacogenomic information of the Zhuang population and refine the study on the differences between the Zhuang population and the other 26 populations. More importantly, this study may provide certain theoretical support for drug use in the Zhuang population. Nonetheless, there are some limitations to our study. Our sample size was relatively small in this study. To design a comprehensive, systematic, disease-specific treatment protocol for the Zhuang population, we need to further expand the sample size for more in-depth studies. In addition, only Agena MassARRAY was used for genotyping in this study, and no other orthogonal method was employed to validate the sequencing, which will be utilized in subsequent studies.

## Conclusion

In short, the genotype frequencies of *CYP3A5* (rs776746), *ACE* (rs4291), *CYP2D6* (rs1065852), and *KCNH2* (rs1805123) showed significant disparities between the Zhuang population and 26 other populations. Our study can not only enrich the pharmacogenomics database of the Zhuang population but also provide a theoretical basis for tailored therapy in this population and ensure safe drug use for patients.

### Supplementary Information

Supplementary Tables.

## Data Availability

The datasets used or analyzed during the current study are available from the corresponding author upon reasonable request.
