# Supplementary material for: Very important pharmacogenetic variants landscape and potential clinical relevance in the Zhuang population from Yunnan province
Source: Sci Rep. 2024 Mar 29;14:7495. doi: 10.1038/s41598-024-58092-w (PMC10980727; doi:10.1038/s41598-024-58092-w)
Supplement: Supplementary file 1 — Supplementary Tables. [file 41598_2024_58092_MOESM1_ESM.docx]

Table S1 Primer sequences for selected SNVs

| SNP ID | 2nd-PCRP | 1st-PCRP | UEP-SEQ | |
| --- | --- | --- | --- | --- |
| rs11572325 | ACGTTGGATGTTGTTGTGCCCAAAGTCAGG | ACGTTGGATGCATTACTTGTTCTGATGCTC | GTCAGGAAATATCAGCTTGA | |
| rs10889160 | ACGTTGGATGGGATAATGAGAGGAAGTTGC | ACGTTGGATGGTAGATTACATCAGATTCC | GGGAGAAAATCGAACTTTGTG | |
| rs890293 | ACGTTGGATGTGGCATCTTCGCAGGGTGCT | ACGTTGGATGTGCTCTTCTGCGGTCCAAG | TGGGCACGGCTGGGAGC | |
| rs1760217 | ACGTTGGATGAAAACCTATTCCCCTGTCTG | ACGTTGGATGCATCTCTCTTGAAACCTGAC | GGTCTGAAATTTAATGCTACACAATG | |
| rs1801159 | ACGTTGGATGTGCGCTAGCAAGACCAAAAG | ACGTTGGATGCTCCTATTGATCTGGTGGAC | GCAAGACCAAAAGGATTTA | |
| rs1801265 | ACGTTGGATGATCCTGGCTTTAAATCCTCG | ACGTTGGATGCTTGTCTAATTTCTTGGCCG | CACAAACTCATGCAACTCTG | |
| rs5275 | ACGTTGGATGCACTGTCGATGTTTCCAATG | ACGTTGGATGTGCACTGATACCTGTTTTTG | AGGTTTGAAATTTTAAAGTACTTTTGGT | |
| rs12139527 | ACGTTGGATGGTTGCCATGATGAAGTTTGC | ACGTTGGATGAAGACCCCACTCAGCCTACT | GTTTGCATCAGCTGCC | |
| rs3850625 | ACGTTGGATGGGAAGTTCTACGCCACATTC | ACGTTGGATGTGGGCCGATAGCCATAATAC | TCCGGAAGTTCATGAAA | |
| rs2306238 | ACGTTGGATGGGGAGTACTGGATAGAGTC | ACGTTGGATGAGATGGGTCTAATCCCTCAG | TTTTAGAGTCCTCTAGTCTAGGT | |
| rs2231142 | ACGTTGGATGTGATGTTGTGATGGGCACTC | ACGTTGGATGGTCATAGTTGTTGCAAGCCG | ATGACGGTGAGAGAAAACTTA | |
| rs2231137 | ACGTTGGATGTCAGGTCATTGGAAGCTGTC | ACGTTGGATGGATGTCTTCCAGTAATGTCG | GCATGGGTGTTTCCTTGTGACA | |
| rs698 | ACGTTGGATGAGAGCGAAGCAGGTCAAATC | ACGTTGGATGTCCCCAAACTTGTGGCTGAC | CTTCATTTATTTTTTCAAAAGGTAAAA | |
| rs776746 | ACGTTGGATGGTAATGTGGTCCAAACAGGG | ACGTTGGATGATGTACCACCCAGCTTAACG | CGCCAAACAGGGAAGAGATA | |
| rs2242480 | ACGTTGGATGTGCTAAGGTTTCACCTCCTC | ACGTTGGATGGCAGGAGGAAATTGATGCAG | TTAACCTCCTCCCTCCTTCTCCATGTA | |
| rs4646244 | ACGTTGGATGTACATACCTCTGGCATGCTG | ACGTTGGATGTTGTAGTCCATCTGCCCAAG | GCATGCTGCCACATGA | |
| rs4271002 | ACGTTGGATGCCCAAAGGTAACACACAATG | ACGTTGGATGGCAGAAACAAAGCCATATGA | AATGCATGTGGTATAAGTGT | |
| rs1041983 | ACGTTGGATGAGACCACAATGTTAGGAGGG | ACGTTGGATGCAGGAGAAGGTGAACCATGC | CAATGTTAGGAGGGTATTTTTA | |
| rs1801280 | ACGTTGGATGCAAATACAGCACTGGCATGG | ACGTTGGATGGACCCAGCATCGACAATGTA | TCCTGCAGGTGACCA | |
| rs1799929 | ACGTTGGATGGGCAGGAGATGAGAATTAAG | ACGTTGGATGTTCTGCTTGACAGAAGAGAG | CTTCGCTCTCCTGATTTGGTCCA | |
| rs1799930 | ACGTTGGATGCCTGCCAAAGAAGAAACACC | ACGTTGGATGAAGATGTTGGAGACGTCTGC | CCAGACTTATTTACGCTTGAACCTC | |
| rs1208 | ACGTTGGATGACAATACAGATCTGGTCGAG | ACGTTGGATGATTTCTCCCCAAGGAAATC | GGTTGAAGAAGTGCTGA | |
| rs1799931 | ACGTTGGATGGGGTGATACATACACAAGGG | ACGTTGGATGGGAAGAGGTTGAAGAAGTGC | CCTTATTCTAAATAGTAAGGGAT | |
| rs1495741 | ACGTTGGATGTCTCTCAGGAAAGGAGCAAA | ACGTTGGATGGGCCTCACATGGTCACTTC | TCTGAAGGATGATTTTCATAATAAT | |
| rs2115819 | ACGTTGGATGTTTGTGTAACACTGGGATGG | ACGTTGGATGGCCACAGGAGATTTTAGTTC | CCATGGGATGGAAAGGGT | |
| rs12248560 | ACGTTGGATGCAAATTTGTGTCTTCTGTTC | ACGTTGGATGGGATTTGAGCTGAGGTCTTC | TTGTGTCTTCTGTTCTCAAAG | |
| rs4244285 | ACGTTGGATGGCAATAATTTTCCCACTATC | ACGTTGGATGTCCATCGATTCTTGGTGTTC | CCCACTATCATTGATTATTTCCC | |
| rs7909236 | ACGTTGGATGTTTCTCCATCATCACAGCAC | ACGTTGGATGGCCAATCTAGGAGATTCTGG | CCCTCACAGCACATTGGAA | |
| rs17110453 | ACGTTGGATGACACTGATTTCCCTCAAGGT | ACGTTGGATGCTGTGATGATGGAGAAACAC | CCTTCCCTCAAGGTCATAAA | |
| rs3813867 | ACGTTGGATGCAACGCCCCTTCTTGGTTCA | ACGTTGGATGGCAAGTCATTGGTTGTGCTG | CCTTCTTGGTTCAGGAGAG | |
| rs6413432 | ACGTTGGATGTCCCAAGTAACTGGGCCACA | ACGTTGGATGCTGTGCCCAGCCAAAATAAT | ACACACCCAGCTGATTAAAAATT | |
| rs2070676 | ACGTTGGATGATCCTTCACTAAGCAACTCC | ACGTTGGATGGAAACCCCCAGTGAAGAATG | AATGCACTAAGCAACTCCTTCAACT | |
| rs5219 | ACGTTGGATGCGTTGCAGTTGCCTTTCTTG | ACGTTGGATGAGGAATACGTGCTGACACGC | CGGTCACGGTACCTGGGCT | |
| rs2306283 | ACGTTGGATGACCTTTTCCCACTATCTCAG | ACGTTGGATGGATGTTCTTACAGTTACAGG | TTGATGTTGAATTTTCTGATGAAT | |
| rs762551 | ACGTTGGATGCTAAGCTCCATCTACCATGC | ACGTTGGATGGAATCTTGAGGCTCCTTTCC | CTACCATGCGTCCTG | |
| rs2472304 | ACGTTGGATGAACCCTATAGCCAGGAGAAG | ACGTTGGATGACACAGCAGGCACATAACAG | CCAGGAGAAGCCTTGA | |
| rs750155 | ACGTTGGATGCATCTCCACTGGGTGGAGG | ACGTTGGATGAGAGTCCAGCTGCACTGAG | GGGTCCCAGCAGGAA | |
| rs1800764 | ACGTTGGATGAAGACAACCCATGGGATGAG | ACGTTGGATGCTTGGAATGTACCCACTGAG | TGCAAAGTATGTACAGCA | |
| rs4291 | ACGTTGGATGCAGAGGAAGCTGGAGAAAGG | ACGTTGGATGTCGGGTGTTCCGGCAAACTG | CCCCGGGCCTCCTCTCTTT | |
| rs4267385 | ACGTTGGATGAGCCAGGTTATCTCTCTAGG | ACGTTGGATGGATTGCACAGCCCATAAGAG | TCTTATAACTTGACCTCTTATGTTCAC | |
| rs2108622 | ACGTTGGATGCTAGGAGCCTTGGAATGGAC | ACGTTGGATGTGCCTCATCAGTGTTTTCGG | CCTCAGGGTCCGGCCACA | |
| rs3093105 | ACGTTGGATGATGTCCCAGCTGAGCCTGT | ACGTTGGATGTGTCATAGAAGGCGTAGGTC | AGACTGTCCTGGCTGGGCCTC | |
| rs8192726 | ACGTTGGATGTCCTCGTCCTGGGTGTTTTC | ACGTTGGATGGGCTTCCTCATCGACGCCC | TCCTTCTCCTGCCCC | |
| rs1051298 | ACGTTGGATGTGGCACATACCAAGGCCAG | ACGTTGGATGCTTCTGAAGTGTGTCCATCC | GAAGGCCAGCACGTCC | |
| rs1051296 | ACGTTGGATGTCTGAAGTGTGTCCATCCTG | ACGTTGGATGATACCAAGGCCAGCACGTC | TAGCTGCTCCCACACT | |
| rs1131596 | ACGTTGGATGACCATCCTGCTCAGGCCAC | ACGTTGGATGATCTTCCAAGGTGCCCTGAC | GGGGACGAAGGTGAC | |
| rs1065852 | ACGTTGGATGTGCTCCTGGTGGACCTGATG | ACGTTGGATGTGGAAGTCCACATGCAGCAG | CCCCTCTGGGCTGCACGCTAC | |
| rs1805123 | ACGTTGGATGGCAAGCGCAAGTTGTCCTTC | ACGTTGGATGTAAAGCAGACACGGCCCAC | | AGCAGCAGGCGCACGGACA |

SNVs: single nucleotide variants.

Table S2 Genotype distribution differences of 26 populations compared with Yunnan Zhuang population

| SNP ID | Genes | EAS | | | | | AFR | | | | | | | AMR |
| --- | --- | --- | --- | --- | --- | --- | --- | --- | --- | --- | --- | --- | --- | --- |
|  |  | CDX | CHB | CHS | JPT | KHV | ACB | ASW | ESN | GWD | LWK | MSL | YRI | CLM |
| rs11572325 | *CYP2J2* | - | 0.376 | - | - | - | **8.15E-06** | **3.82E-05** | **1.96E-05** | **5.08E-05** | **1.51E-04** | **0.001** | **4.14E-05** | **0.024** |
| rs10889160 | *CYP2J2* | 0.602 | **0.014** | **0.048** | **2.54E-04** | 0.201 | **1.47E-19** | **9.71E-11** | **2.94E-22** | **8.02E-16** | **8.45E-17** | **1.82E-19** | **9.11E-22** | 0.304 |
| rs890293 | *CYP2J2* | - | 0.338 | - | - | - | **0.001** | **1.17E-06** | **8.44E-10** | **1.99E-07** | **1.56E-06** | **2.57E-11** | **3.43E-07** | - |
| rs1760217 | *DPYD* | 0.054 | 0.391 | 0.306 | **0.002** | 0.978 | 0.091 | 0.697 | 0.612 | **0.007** | 0.093 | 0.436 | **0.020** | **0.037** |
| rs1801159 | *DPYD* | 0.147 | 0.066 | **0.001** | **0.038** | 0.191 | **2.47E-07** | **6.90E-05** | **8.52E-05** | **7.27E-13** | 0.360 | **2.49E-11** | **4.31E-07** | **1.49E-04** |
| rs1801265 | *DPYD* | **0.012** | - | - | **0.028** | - | **1.64E-14** | **7.54E-19** | **1.56E-18** | **1.82E-21** | **3.06E-22** | **1.85E-14** | **1.34E-19** | **2.30E-05** |
| rs5275 | *PTGS2* | 0.947 | 0.088 | 0.452 | 0.546 | 0.780 | **9.28E-19** | **2.50E-12** | **6.19E-23** | **5.67E-18** | **6.59E-19** | **1.92E-21** | **3.04E-23** | **5.31E-05** |
| rs12139527 | *CACNA1S* | 0.998 | 0.416 | 0.928 | 0.723 | 0.509 | **1.10E-23** | **1.42E-19** | **1.26E-31** | **3.10E-34** | **1.04E-26** | **1.08E-30** | **7.57E-31** | 0.451 |
| rs3850625 | *CACNA1S* | 0.417 | **0.004** | 0.353 | 0.762 | 0.103 | 0.774 | 0.503 | 0.131 | 0.098 | 0.131 | 0.174 | 0.109 | **1.52E-05** |
| rs2306238 | *RYR2* | 0.160 | 0.253 | 0.212 | 0.128 | 0.634 | **1.70E-05** | **0.004** | **6.93E-08** | **1.02E-06** | **4.01E-06** | **4.26E-07** | **2.66E-06** | 0.133 |
| rs2231142 | *ABCG2* | 0.784 | **0.014** | 0.284 | **0.003** | **0.001** | **1.30E-09** | **0.004** | **3.59E-11** | **1.42E-10** | **3.59E-11** | **5.40E-08** | **5.17E-12** | 0.063 |
| rs2231137 | *ABCG2* | 0.093 | 0.090 | 0.387 | **2.13E-07** | 0.907 | **3.27E-17** | **1.40E-14** | **2.72E-17** | **2.11E-20** | **7.43E-11** | **6.58E-12** | **1.04E-17** | **2.06E-08** |
| rs698 | *ADH1C* | 0.577 | **0.005** | **0.043** | **0.048** | 0.146 | 0.336 | 0.409 | **0.044** | 0.201 | 0.946 | 0.361 | **0.048** | **0.001** |
| rs776746 | *CYP3A5* | **8.15E-22** | **5.32E-20** | **1.62E-19** | **5.81E-22** | **6.45E-22** | **2.28E-43** | **5.81E-38** | **9.74E-50** | **1.68E-46** | **7.50E-47** | **2.72E-45** | **2.35E-51** | **1.03E-13** |
| rs2242480 | *CYP3A4* | 0.715 | 0.415 | 0.420 | 0.540 | 0.660 | **9.64E-26** | **1.65E-17** | **4.74E-38** | **7.33E-31** | **2.10E-38** | **1.13E-34** | **9.72E-33** | 0.978 |
| rs4646244 | *NAT2* | 0.456 | 0.936 | 0.162 | 0.078 | **2.04E-04** | 0.214 | 0.243 | 0.159 | 0.909 | 0.480 | 0.215 | 0.991 | 0.279 |
| rs4271002 | *NAT2* | **2.87E-04** | 0.134 | 0.116 | 0.153 | 0.351 | 0.253 | 0.649 | **9.08E-05** | **2.06E-04** | 0.091 | 0.316 | 0.072 | 0.185 |
| rs1041983 | *NAT2* | **0.002** | 0.988 | 0.068 | 0.248 | **7.78E-05** | **0.005** | 0.148 | **1.35E-04** | 0.613 | 0.206 | **0.001** | **0.003** | 0.220 |
| rs1801280 | *NAT2* | **0.044** | 0.382 | 0.146 | 0.715 | 0.065 | **4.85E-19** | **4.86E-19** | **1.62E-19** | **2.96E-24** | **1.05E-27** | **5.47E-16** | **5.09E-16** | **9.78E-27** |
| rs1799929 | *NAT2* | 0.077 | 0.498 | 0.224 | 0.762 | 0.201 | **6.55E-15** | **6.25E-15** | **6.18E-13** | **3.38E-20** | **1.00E-23** | **9.37E-12** | **3.67E-09** | **1.12E-24** |
| rs1799930 | *NAT2* | 0.756 | 0.655 | 0.441 | 0.163 | **3.97E-04** | 0.339 | 0.141 | 0.200 | 0.499 | 0.197 | 0.381 | 0.923 | 0.338 |
| rs1208 | *NAT2* | 0.077 | 0.498 | 0.132 | 0.762 | 0.058 | **2.31E-25** | **7.37E-22** | **4.83E-27** | **9.05E-33** | **1.32E-32** | **4.64E-24** | **2.36E-27** | **1.05E-26** |
| rs1799931 | *NAT2* | **0.001** | 0.137 | 0.326 | 0.098 | 0.411 | **3.98E-04** | **0.018** | **4.30E-06** | **1.84E-06** | **1.26E-06** | **0.004** | **1.98E-04** | **0.024** |
| rs1495741 | *NAT2* | **2.03E-04** | 0.908 | **0.014** | 0.509 | **7.39E-06** | **1.42E-05** | **1.05E-07** | **2.29E-04** | **5.63E-05** | **3.17E-09** | **0.002** | **0.007** | **3.46E-12** |
| rs2115819 | *ALOX5* | **0.002** | **1.09E-04** | **0.040** | **0.003** | 0.051 | **1.49E-38** | **8.51E-26** | **4.34E-38** | **4.74E-42** | **7.49E-34** | **9.70E-33** | **6.76E-41** | **4.00E-17** |
| rs12248560 | *CYP2C19* | - | - | - | - | - | **4.50E-25** | **1.48E-17** | **7.51E-22** | **1.18E-21** | **1.46E-15** | **7.69E-23** | **4.81E-21** | **2.28E-11** |
| rs4244285 | *CYP2C19* | 0.606 | 0.197 | 0.100 | 0.800 | 0.905 | **0.001** | **0.002** | 0.054 | **1.47E-05** | 0.077 | **0.010** | **0.001** | **2.61E-06** |
| rs7909236 | *CYP2C8* | 0.620 | 0.769 | 0.102 | 0.158 | **0.011** | **0.004** | 0.454 | **1.43E-06** | **2.42E-07** | **6.42E-05** | **8.66E-06** | **4.56E-07** | **1.26E-06** |
| rs17110453 | *CYP2C8* | 0.927 | 0.100 | **0.026** | **0.005** | 0.575 | **1.75E-13** | **1.36E-09** | **7.43E-14** | **5.84E-17** | **3.10E-15** | **1.82E-13** | **1.21E-15** | **8.33E-06** |
| rs3813867 | *CYP2E1* | 0.779 | **0.003** | 0.140 | 0.282 | 0.067 | **0.007** | 0.131 | 0.081 | 0.056 | **5.46E-05** | **0.023** | 0.097 | 0.820 |
| rs6413432 | *CYP2E1* | **1.81E-06** | **3.90E-07** | **7.62E-06** | **7.17E-06** | **2.88E-07** | - | - | 0.119 | **0.031** | - | **0.020** | 0.237 | **0.039** |
| rs2070676 | *CYP2E1* | **0.021** | 0.266 | 0.677 | 0.423 | 0.058 | **1.78E-25** | **1.45E-13** | **1.04E-24** | **3.05E-26** | **8.00E-31** | **2.96E-26** | **3.50E-24** | 0.073 |
| rs5219 | *KCNJ11* | **1.13E-04** | **0.001** | 0.230 | **0.013** | **0.034** | **2.98E-19** | **6.49E-09** | **5.01E-28** | **3.94E-29** | **3.34E-27** | **3.40E-25** | **8.78E-30** | **8.52E-07** |
| rs2306283 | *SLCO1B1* | 0.082 | 0.287 | 0.086 | 0.207 | 0.153 | 0.086 | 0.650 | **6.16E-05** | **0.036** | **0.004** | 0.082 | **0.023** | **1.48E-06** |
| rs762551 | *CYP1A2* | 0.302 | **0.041** | 0.136 | **0.006** | 0.832 | **0.013** | 0.219 | **3.48E-06** | **0.001** | **4.53E-08** | **0.002** | **2.86E-05** | 0.321 |
| rs2472304 | *CYP1A2* | 0.380 | 0.969 | 0.170 | **0.008** | 0.187 | 0.205 | 0.568 | **7.35E-06** | **4.86E-05** | **8.28E-05** | **4.17E-05** | **8.77E-06** | **3.48E-12** |
| rs750155 | *SULT1A1* | 0.385 | **5.03E-05** | 0.596 | **3.47E-06** | 0.075 | **0.002** | **1.01E-04** | **8.71E-10** | **9.53E-13** | **8.18E-06** | **1.42E-06** | **6.52E-08** | 0.072 |
| rs1800764 | *ACE* | 0.587 | 0.085 | 0.573 | 0.175 | 0.068 | **1.07E-26** | **4.95E-17** | **4.17E-34** | **6.98E-40** | **4.78E-29** | **5.98E-37** | **2.54E-39** | 0.080 |
| rs4291 | *ACE* | **2.44E-17** | **9.38E-22** | **7.87E-18** | **4.20E-15** | **1.50E-21** | **5.93E-16** | **4.76E-15** | **4.06E-17** | **1.65E-14** | **3.91E-21** | **1.36E-13** | **4.08E-22** | **2.53E-16** |
| rs4267385 | *ACE* | 0.548 | 0.987 | 0.413 | 0.580 | 0.412 | **5.38E-26** | **1.54E-16** | **1.35E-27** | **1.57E-33** | **7.19E-35** | **1.15E-31** | **1.41E-31** | **9.42E-07** |
| rs2108622 | *CYP4F2* | 0.623 | 0.352 | 0.696 | **0.003** | **0.042** | **0.010** | **0.041** | **4.11E-06** | **1.84E-04** | 0.058 | **0.047** | **1.65E-05** | **0.004** |
| rs3093105 | *CYP4F2* | - | - | - | **2.56E-59** | - | **1.15E-37** | **1.86E-30** | **5.69E-33** | **2.44E-41** | **7.01E-38** | **2.46E-36** | **3.69E-35** | **5.66E-44** |
| rs8192726 | *CYP2A6* | 0.464 | 0.225 | **0.044** | 0.115 | 0.061 | **1.18E-04** | **0.043** | **0.010** | **3.55E-05** | **0.006** | **0.001** | **0.005** | **3.04E-06** |
| rs1051298 | *SLC19A1* | 0.075 | 0.073 | 0.398 | 0.065 | **0.035** | **0.004** | 0.187 | 0.573 | **1.26E-04** | **0.046** | 0.054 | 0.149 | 0.097 |
| rs1051296 | *SLC19A1* | **0.011** | **0.001** | 0.275 | **0.004** | **0.011** | **0.050** | **0.003** | **0.001** | **0.002** | **1.82E-04** | **0.034** | **0.011** | **0.003** |
| rs1131596 | *SLC19A1* | **0.049** | **0.017** | 0.756 | **0.013** | 0.289 | **3.32E-09** | **0.024** | **0.002** | **3.17E-14** | **1.37E-10** | **3.85E-09** | **2.32E-08** | **0.022** |
| rs1065852 | *CYP2D6* | **0.001** | **0.030** | **0.001** | **5.47E-07** | **0.005** | **7.31E-23** | **4.59E-18** | **8.94E-30** | **1.34E-28** | **8.17E-37** | **3.59E-20** | **9.51E-29** | **1.66E-19** |
| rs1805123 | *KCNH2* | **1.30E-51** | **3.59E-60** | **4.17E-60** | **3.57E-59** | **3.69E-53** | **4.89E-62** | **6.05E-51** | **1.44E-64** | **1.08E-66** | **1.29E-63** | **1.58E-61** | **1.60E-66** | **1.41E-42** |

Table S2 Genotype distribution differences of 26 populations compared with Yunnan Zhuang population (continue)

| SNP ID | Genes | AMR | | | EUR | | | | | SAS | | | | |
| --- | --- | --- | --- | --- | --- | --- | --- | --- | --- | --- | --- | --- | --- | --- |
|  |  | MXL | PEL | PUR | CEU | FIN | GBR | IBS | TSI | BEB | GIH | ITU | PJL | STU |
| rs11572325 | *CYP2J2* | - | - | **7.89E-05** | **0.039** | **4.74E-04** | 0.158 | **0.044** | **0.049** | - | - | 0.372 | - | - |
| rs10889160 | *CYP2J2* | 0.458 | 0.407 | **1.48E-04** | 0.382 | **1.34E-05** | 0.341 | **0.016** | 0.179 | 0.785 | 0.157 | 0.203 | 0.859 | 0.221 |
| rs890293 | *CYP2J2* | - | - | 0.099 | - | **0.045** | 0.252 | - | - | - | - | 0.138 | - | - |
| rs1760217 | *DPYD* | 0.819 | 0.206 | **0.002** | 0.862 | **0.008** | **0.013** | **0.026** | 0.066 | 0.184 | 0.671 | **1.07E-05** | **0.004** | **4.81E-05** |
| rs1801159 | *DPYD* | **0.020** | **0.044** | **2.76E-04** | **3.12E-06** | **2.63E-07** | **3.21E-05** | **0.001** | **0.009** | **2.43E-09** | **4.13E-10** | **4.57E-15** | **1.80E-10** | **4.52E-13** |
| rs1801265 | *DPYD* | **9.52E-06** | **0.013** | **1.00E-06** | **0.024** | **2.85E-09** | **0.008** | **1.50E-04** | **6.20E-05** | **0.003** | **1.16E-11** | **4.66E-10** | **6.11E-09** | **0.001** |
| rs5275 | *PTGS2* | **0.005** | **3.84E-05** | **0.017** | **8.01E-05** | 0.457 | 0.099 | **0.007** | 0.053 | **7.24E-05** | **1.07E-04** | **0.001** | **5.02E-09** | **1.52E-05** |
| rs12139527 | *CACNA1S* | **0.021** | 0.170 | **0.017** | 0.752 | 0.916 | 0.626 | 0.879 | 0.877 | **0.030** | **0.006** | 0.575 | 0.337 | 0.355 |
| rs3850625 | *CACNA1S* | **1.91E-04** | **6.07E-05** | 0.127 | **0.001** | **3.04E-11** | **1.07E-07** | **0.001** | **3.77E-05** | **2.24E-08** | **1.17E-19** | **2.68E-12** | **4.27E-11** | **7.10E-09** |
| rs2306238 | *RYR2* | **0.004** | **0.001** | **0.021** | 0.531 | 0.301 | 0.794 | 0.827 | 0.499 | 0.154 | 0.358 | **0.012** | **0.006** | **0.003** |
| rs2231142 | *ABCG2* | 0.958 | 0.280 | **0.010** | **0.030** | **0.002** | 0.155 | **9.27E-05** | **1.28E-05** | 0.066 | **8.87E-05** | **0.010** | **0.009** | **0.001** |
| rs2231137 | *ABCG2* | **0.001** | 0.237 | **1.38E-08** | **3.39E-19** | **4.61E-13** | **2.17E-18** | **4.02E-18** | **5.28E-15** | **0.002** | **8.91E-09** | **1.42E-12** | **1.07E-11** | **1.12E-05** |
| rs698 | *ADH1C* | **2.91E-04** | 0.354 | **2.54E-10** | **1.08E-17** | **5.94E-19** | **6.67E-14** | **9.12E-07** | **9.77E-07** | 0.302 | **2.55E-05** | **0.002** | **1.37E-07** | **2.15E-08** |
| rs776746 | *CYP3A5* | **1.45E-17** | **1.19E-09** | **1.15E-15** | **1.61E-05** | **7.01E-06** | **1.47E-06** | **2.30E-07** | **1.23E-06** | **5.72E-24** | **5.31E-21** | **2.07E-22** | **1.10E-21** | **2.70E-22** |
| rs2242480 | *CYP3A4* | 0.068 | **1.40E-09** | 0.137 | **5.05E-10** | **1.86E-08** | **2.29E-08** | **4.78E-06** | **5.34E-08** | 0.088 | 0.479 | 0.060 | **0.010** | 0.130 |
| rs4646244 | *NAT2* | 0.122 | **0.004** | 0.801 | **0.003** | 0.067 | **0.018** | **0.042** | 0.072 | 0.210 | **2.12E-05** | **4.70E-04** | **4.15E-04** | **9.95E-07** |
| rs4271002 | *NAT2* | 0.535 | **0.015** | 0.200 | **0.027** | 0.584 | 0.779 | 0.775 | 0.647 | 0.241 | 0.930 | 0.415 | 0.909 | 0.788 |
| rs1041983 | *NAT2* | 0.185 | **0.005** | 0.184 | 0.072 | 0.224 | 0.098 | 0.480 | 0.269 | 0.870 | 0.112 | 0.289 | 0.201 | **0.003** |
| rs1801280 | *NAT2* | **4.50E-24** | **1.34E-20** | **1.78E-27** | **3.56E-31** | **2.53E-33** | **5.89E-33** | **1.73E-36** | **2.44E-32** | **1.69E-24** | **5.68E-24** | **1.80E-25** | **7.16E-31** | **1.41E-20** |
| rs1799929 | *NAT2* | **1.84E-22** | **3.15E-19** | **1.08E-23** | **2.04E-30** | **3.87E-31** | **1.07E-30** | **1.03E-35** | **1.30E-31** | **2.11E-22** | **7.73E-21** | **7.06E-23** | **1.13E-26** | **9.82E-19** |
| rs1799930 | *NAT2* | 0.148 | **0.001** | 0.782 | **0.007** | 0.233 | **0.019** | **0.031** | 0.097 | 0.263 | **1.11E-05** | **0.001** | **2.15E-04** | **2.54E-07** |
| rs1208 | *NAT2* | **6.69E-28** | **7.16E-20** | **9.13E-27** | **4.93E-29** | **1.85E-30** | **1.02E-30** | **9.31E-36** | **2.80E-32** | **1.17E-26** | **2.75E-23** | **3.12E-24** | **7.53E-31** | **2.92E-22** |
| rs1799931 | *NAT2* | 0.479 | **0.030** | 0.073 | **3.37E-07** | **2.68E-04** | **4.28E-05** | **9.27E-05** | **4.32E-06** | 0.242 | **0.004** | **0.007** | **0.008** | 0.055 |
| rs1495741 | *NAT2* | **1.12E-07** | **0.004** | **7.38E-11** | **1.93E-13** | **1.66E-15** | **8.63E-16** | **6.88E-20** | **6.96E-14** | **1.18E-12** | **1.13E-17** | **3.28E-15** | **6.09E-21** | **2.66E-19** |
| rs2115819 | *ALOX5* | **1.53E-11** | **2.59E-07** | **1.26E-15** | **4.32E-25** | **3.56E-20** | **3.24E-19** | **6.08E-21** | **7.28E-22** | **9.82E-17** | **4.99E-24** | **1.42E-21** | **1.00E-16** | **2.89E-15** |
| rs12248560 | *CYP2C19* | **5.71E-09** | - | **7.65E-16** | **1.15E-20** | **1.68E-19** | **2.79E-21** | **1.33E-19** | **1.33E-19** | - | **7.28E-12** | **5.54E-12** | **3.36E-12** | **1.76E-11** |
| rs4244285 | *CYP2C19* | **4.23E-04** | **2.99E-09** | **2.39E-05** | **4.15E-05** | 0.058 | **3.32E-04** | **9.91E-05** | **6.12E-08** | 0.702 | 0.235 | 0.157 | 0.095 | **0.018** |
| rs7909236 | *CYP2C8* | **1.27E-05** | **8.08E-10** | 0.070 | **4.53E-05** | **3.35E-05** | **0.006** | 0.071 | 0.132 | **0.010** | **0.001** | **0.010** | **0.016** | 0.227 |
| rs17110453 | *CYP2C8* | **0.001** | **1.36E-08** | **6.18E-05** | **2.48E-06** | 0.273 | **3.68E-05** | 0.189 | **9.12E-06** | 0.105 | 0.094 | 0.158 | 0.369 | **0.017** |
| rs3813867 | *CYP2E1* | 0.832 | 0.780 | **0.013** | **0.013** | **3.79E-04** | **1.63E-04** | **1.83E-05** | **0.003** | **1.20E-05** | **1.00E-06** | **1.16E-06** | **2.78E-06** | **3.11E-07** |
| rs6413432 | *CYP2E1* | 0.114 | **0.008** | **0.015** | 0.361 | **0.015** | 0.060 | 0.078 | 0.335 | **0.002** | **3.46E-06** | **0.003** | 0.106 | **0.002** |
| rs2070676 | *CYP2E1* | 0.299 | 0.113 | **0.020** | 0.074 | **0.005** | **0.024** | 0.253 | 0.051 | 0.578 | 0.322 | 0.705 | 0.492 | 0.886 |
| rs5219 | *KCNJ11* | 0.103 | 0.106 | **3.07E-04** | 0.384 | **0.037** | **0.004** | **0.018** | **0.001** | **0.002** | **0.001** | **0.013** | **2.17E-06** | **0.005** |
| rs2306283 | *SLCO1B1* | **7.78E-10** | **3.53E-07** | **1.09E-04** | **8.99E-11** | **5.18E-09** | **5.74E-13** | **2.70E-11** | **1.20E-13** | **0.004** | **0.001** | **0.022** | **9.27E-08** | **0.001** |
| rs762551 | *CYP1A2* | **0.029** | **2.17E-04** | 0.842 | 0.503 | 0.255 | 0.321 | 0.054 | **0.022** | **0.001** | **5.13E-06** | **2.96E-05** | **5.70E-05** | **1.26E-07** |
| rs2472304 | *CYP1A2* | **1.01E-04** | 0.989 | **2.22E-19** | **1.27E-31** | **3.26E-24** | **3.18E-30** | **2.90E-28** | **7.29E-22** | 0.084 | 0.234 | 0.790 | **0.001** | 0.921 |
| rs750155 | *SULT1A1* | 0.218 | **1.17E-12** | **6.26E-05** | **0.004** | 0.330 | **0.005** | **0.002** | **3.76E-04** | **3.83E-12** | **6.89E-08** | **6.20E-11** | **2.10E-08** | **1.25E-17** |
| rs1800764 | *ACE* | 0.129 | **0.001** | 0.079 | **0.011** | 0.172 | 0.114 | 0.384 | **0.001** | 0.685 | 0.147 | 0.210 | 0.580 | 0.949 |
| rs4291 | *ACE* | **3.27E-19** | **2.23E-26** | **9.16E-16** | **1.28E-17** | **1.51E-12** | **2.50E-18** | **1.41E-15** | **8.52E-19** | **3.55E-16** | **6.16E-16** | **5.81E-22** | **5.34E-17** | **6.88E-16** |
| rs4267385 | *ACE* | **3.89E-04** | 0.875 | **4.81E-10** | **3.57E-11** | **2.23E-10** | **1.49E-12** | **1.72E-13** | **3.89E-22** | 0.074 | **4.20E-04** | 0.131 | **2.91E-04** | **0.004** |
| rs2108622 | *CYP4F2* | 0.188 | 0.123 | **0.002** | **0.016** | 0.345 | **0.001** | **3.00E-06** | **3.05E-06** | **8.50E-09** | **7.07E-11** | **6.54E-09** | **5.88E-08** | **5.84E-10** |
| rs3093105 | *CYP4F2* | **1.39E-42** | **1.35E-55** | **1.54E-44** | **1.21E-44** | **1.84E-49** | - | **7.51E-35** | **1.34E-39** | **6.52E-45** | **1.47E-46** | **1.85E-42** | **3.34E-42** | **2.80E-46** |
| rs8192726 | *CYP2A6* | **3.72E-04** | **4.46E-05** | **3.48E-06** | **2.15E-05** | 0.090 | **6.09E-06** | **1.27E-05** | **1.38E-04** | 0.096 | 0.213 | **0.019** | 0.356 | **0.019** |
| rs1051298 | *SLC19A1* | **2.48E-05** | **1.15E-04** | 0.092 | **0.005** | 0.083 | **3.25E-04** | **0.008** | **0.023** | 0.196 | **0.029** | 0.205 | 0.342 | **0.021** |
| rs1051296 | *SLC19A1* | **6.24E-08** | **9.47E-08** | **0.001** | **7.75E-05** | **0.002** | **1.09E-06** | **1.71E-04** | **1.69E-04** | 0.092 | **0.025** | **0.002** | **0.003** | **0.003** |
| rs1131596 | *SLC19A1* | **4.37E-07** | **1.21E-05** | **0.006** | **0.045** | **0.022** | **2.49E-06** | **0.001** | **0.003** | **0.002** | **0.001** | **4.88E-04** | **2.09E-04** | **0.001** |
| rs1065852 | *CYP2D6* | **6.20E-18** | **8.28E-30** | **1.30E-21** | **8.70E-14** | **4.41E-23** | **5.54E-14** | **4.52E-21** | **8.40E-18** | **1.55E-13** | **1.24E-22** | **1.76E-20** | **4.59E-27** | **3.85E-23** |
| rs1805123 | *KCNH2* | **6.73E-41** | **3.21E-51** | **3.55E-41** | **3.82E-40** | **1.03E-44** | **4.00E-36** | **8.87E-38** | **9.24E-37** | **4.67E-34** | **3.55E-39** | **1.83E-37** | **3.46E-42** | **7.87E-42** |

Bolded font indicates significant results.

Abbreviations: EAS, East Asian; SAS, South Asian; EUR, European; AFR, African; AMR, American; CDX, Chinese Dai in Xishuangbanna, China; CHB, Han Chinese in Beijing, China; CHS, Southern Han Chinese, China; JPT, Japanese in Tokyo, Japan; KHV, Kinh in Ho Chi Minh City; Vietnam; BEB, Bengali in Bangladesh; GIH, Gujarati Indian in Houston, Texas; ITU, Indian Telugu in the UK; PJL, Punjabi in Lahore, Pakistan; STU, Sri Lankan Tamil in the UK; CEU, Western European ancestry; FIN, Finnish in Finland; GBR, British in England and Scotland; IBS, Iberian populations in Spain; TSI, Toscani in Italy; ACB, African Caribbeans in Barbados; ASW, African Ancestry in Southwest USA; ESN, Esan in Nigeria; GWD, Gambian in Western Divisions, The Gambia; LWK, Luhya in Webuye, Kenya; MSL, Mende in Sierra Leone; YRI, Yoruba in Ibadan, Nigeria; CLM, Colombian in Medellin, Colombia; MXL, Mexican Ancestry in Los Angeles, Colombia; PEL, Peruvian in Lima, Peru; PUR, Puerto Rican in Puerto Rico.
